# The mouse *Jhy* gene regulates ependymal cell differentiation and ciliogenesis

**DOI:** 10.1371/journal.pone.0184957

**Published:** 2017-12-06

**Authors:** Hilmarie Muniz-Talavera, Jennifer V. Schmidt

**Affiliations:** Department of Biological Sciences, University of Illinois at Chicago S. Ashland Ave., Chicago, IL, United States of America; National Eye Institute, UNITED STATES

## Abstract

During the first postnatal week of mouse development, radial glial cells lining the ventricles of the brain differentiate into ependymal cells, undergoing a morphological change from pseudostratified cuboidal cells to a flattened monolayer. Concomitant with this change, multiple motile cilia are generated and aligned on each nascent ependymal cell. Proper ependymal cell development is crucial to forming the brain tissue:CSF barrier, and to the establishment of ciliary CSF flow, but the mechanisms that regulate this differentiation event are poorly understood. The *Jhy*^*lacZ*^ mouse line carries an insertional mutation in the *Jhy* gene (formerly *4931429I11Rik*), and homozygous *Jhy*^*lacZ/lacZ*^ mice develop a rapidly progressive juvenile hydrocephalus, with defects in ependymal cilia morphology and ultrastructure. Here we show that beyond just defective motile cilia, *Jhy*^*lacZ/lacZ*^ mice display abnormal ependymal cell differentiation. Ventricular ependyma in *Jhy*^*lacZ/lacZ*^ mice retain an unorganized and multi-layered morphology, representative of undifferentiated ependymal (radial glial) cells, and they show altered expression of differentiation markers. Most *Jhy*^*lacZ/lacZ*^ ependymal cells do eventually acquire some differentiated ependymal characteristics, suggesting a delay, rather than a block, in the differentiation process, but ciliogenesis remains perturbed. *Jhy*^*lacZ/lacZ*^ ependymal cells also manifest disruptions in adherens junction formation, with altered N-cadherin localization, and have defects in the polarized organization of the apical motile cilia that do form. Functional studies showed that cilia of *Jhy*^*lacZ/lacZ*^ mice have severely reduced motility, a potential cause for the development of hydrocephalus. This work shows that JHY does not only control ciliogenesis, but is a crucial component of the ependymal differentiation process, with ciliary defects likely a consequence of altered ependymal differentiation.

## Introduction

The ependyma is a monolayer of multiciliated epithelial cells that lines the ventricles of the vertebrate brain [1]. Ependymal cells serve as a protective barrier between the cerebrospinal fluid (CSF) and the brain tissue, and they are believed to contribute to CSF flow through the ventricular system by the coordinated beating of their apical motile cilia [2–4]. The ependyma produces a small amount of CSF (the majority of the CSF is secreted by the choroid plexus), but paradoxically also absorbs CSF, and provides metabolic support to developing neural stem cells [5,6]. Mouse models with loss of ependymal ciliary motility often develop hydrocephalus, a pathologic increase in ventricular CSF volume, presumably because ciliary stasis reduces both CSF flow and its absorption [7–10]. Mutations in the Hydin gene, for example, cause the production of ependymal cilia that are structurally normal, but are immotile due to microtubule defects [11,12]. Hydin mutant animals develop outwardly visible hydrocephalus within the first postnatal week, and die by 7 weeks of age [13].

Ependymal cells are postmitotic cells that develop from radial glia, a precursor that also gives rise to neurons, astrocytes, and oligodendrocytes [6,14–16]. The terms maturation and differentiation are often used interchangeably to refer to the transition from a radial glial cell to a multiciliated ependymal cell. The Gene Ontology consortium defines differentiation as “the process whereby a relatively unspecialized cell acquires specialized features of a specific cell type”, and maturation as “a developmental process, independent of morphogenetic (shape) change, that is required for a cell to attain its fully functional state”. As the ependymal transition involves clear changes in cell morphology, we will use the term differentiation here to describe this process [17]. The transition from radial glia to ependymal cells occurs during the first postnatal week in the mouse, with the differentiation process moving as a wave across the ventricular surfaces in a caudo-rostral/ventro-dorsal/latero-medial gradient [2,15]. As radial glia differentiate to ependyma, they undergo a morphological change from pseudostratified cuboidal cells to a monolayer of flattened multiciliated cells, and become competent to produce motile cilia. The factors that regulate the transition from radial glial cells to differentiated ependymal cells are not well characterized, and many of the steps in motile ciliogenesis are unknown. FOXJ1 is one protein required for ependymal differentiation and motile cilia formation, and is thought to be at the top of a transcriptional hierarchy controlling these processes. Mice lacking *FoxJ1* have immature ependymal cells that lack cilia, and these animals die in the early postnatal period from multiple abnormalities including hydrocephalus [18–20]. The homeobox gene *Six3* is known to repress radial glial properties, therefore promoting differentiation, and its disruption in mice results in ventricular walls lined with cells that display a mixture of radial glial and ependymal characteristics [8].

As ependymal cells differentiate, they undergo massive replication of centrioles to generate the basal bodies required for multiciliogenesis. Basal bodies arise centrally within the cell, but migrate to one end through processes controlled by the planar cell polarity (PCP) pathway. This basal body localization, termed translational polarity, is uniform in direction across the ventricular surface. Each basal body gives rise to a single cilium, with roughly 40 cilia formed per ependymal cell [21]. These motile cilia are composed of microtubule polymers in a 9+2 arrangement, with 9 outer doublets surrounding a central pair of singlet microtubules. The orientation of the central pair determines the direction of bending of the cilia, and this orientation must be coordinated across the tissue surface for proper CSF flow. Ciliogenesis progresses in a caudo-rostral/ventro-dorsal/medio-lateral gradient as the ependymal cells differentiate, with caudoventral regions of the lateral ventricles carrying abundant cilia by postnatal day 5 (P5), while rostral and dorsal regions become ciliated later [15,22]. Ependymal differentiation and ciliogenesis is completed throughout the ventricular system by P21 [23,24]. Ciliary abnormalities in humans and animal models range from a complete lack of cilia [19,25], malformation of the cilia [10,26], disruption of the axonemal structure [11,27], or the loss of ciliary motility, with or without morphological abnormalities [28]. Loss of ciliary motility can result from altered microtubule organization [29], defects in structural components such as radial spokes or dynein arms [30,31], or changes in translational polarity that impact the direction of ciliary beating [32–34].

We reported previously that a loss of function mutation for the mouse *Jhy* gene (*Jhy*^*lacZ*^) causes congenital hydrocephalus that manifests by P1.5, and leads to death by 6–8 weeks of age [22]. Little is known about the *Jhy* gene, which was unstudied prior to our mapping of the *Jhy*^*lacZ*^ integration. The *Jhy* sequence is conserved across vertebrates, but it has no identifiable paralogs, nor does it contain any recognizable functional domains. As much effort has not yielded an antibody that is competent for immunofluorescence, the cellular localization of the JHY protein remains unknown. Homozygous *Jhy*^*lacZ/lacZ*^ mice develop fewer and shorter ependymal cilia, and these cilia show loss of the central pair of microtubules. These data indicated that *JHY* is required for proper ependymal ciliogenesis, but beyond this preliminary information, how *Jhy* might regulate ciliogenesis was unknown. We now show that in addition to its role in ciliary morphogenesis, *Jhy* is required for proper differentiation of radial glia to functional ependymal cells. In this manuscript, we build on our previous work [22] to show that 1) loss of *Jhy* causes defects not just in ciliary microtubule organization, but in the process of ependymal differentiation itself—ependymal cells of *Jhy*^*lacZ/lacZ*^ mice are delayed in their morphological differentiation, with continued expression of a radial glial cell marker, 2) *Jhy*^*lacZ/lacZ*^ ependyma have defects in ciliary translational and rotational polarity, aspects of epithelial organization regulated by the planar cell polarity (PCP) pathway, 3) *Jhy*^*lacZ/lacZ*^ ependymal cells display abnormal adherens junctions, with mislocalization of N-cadherin and β-catenin proteins, and 4) *Jhy*^*lacZ/lacZ*^ cilia are nearly entirely immotile (previously suggested but not proven), providing a likely mechanism for the *Jhy*^*lacZ*^ hydrocephalus. *Jhy* therefore plays a role in the differentiation and functional specialization of ependymal cells, likely through structural (i.e. adherens junctions) and gene expression changes that may be mediated by N-cadherin and β-catenin.

## Materials & methods

### Animal maintenance

The generation of the *Jhy*^*lacZ*^ mouse line has been previously described [22]. Briefly, *Jhy*^*lacZ*^ was generated by a transgenic insertion on the FVB/N genetic background. The mice were maintained as heterozygotes by crossing to wild type FVB/N animals, then *Jhy*^*lacZ/+*^ mice were intercrossed to generate *Jhy*^*lacZ/lacZ*^ and wild type littermate animals for analysis. Animals were sacrificed using carbon dioxide followed by cervical dislocation as directed by the Office of Animal Care and Institutional Biosafety (OACIB). Genotyping of animals was performed as previously described [22]. This study was carried out in strict accordance with the recommendations in the Guide for the Care and Use of Laboratory Animals of the National Institutes of Health. The protocol was approved by the Office of Animal Care and Institutional Biosafety (OACIB) within the Office of the Vice Chancellor for Research at the University of Illinois at Chicago (Protocol #15–098).

### Histology

Mouse brains at P5, P10 and P14 were dissected and fixed in Bouin’s fixative for 18–24 hours at room temperature (RT). Brains were rinsed in tap water followed by washes in 70% ethanol with saturated lithium carbonate (Sigma-Aldrich, St. Louis, MO). Tissue was then dehydrated in a graded series of ethanol washes, embedded in paraffin and sectioned into 8–10 μm sections using a Leica Microsystems RM2125 microtome (Leica, Wetzlar, Germany). All sections shown are coronal unless stated otherwise. Paraffin was removed though xylene washes and sections rehydrated in a series of ethanol washes. Brain sections were stained using Harris hematoxylin and alcoholic eosin Y solution (Sigma-Aldrich, St. Louis, MO). Slides were subsequently dehydrated, washed in xylene and mounted using Cytoseal XYL (Fisher Scientific, Pittsburgh, PA). Imaging was performed using a Leica MZFLIII dissecting microscope equipped with a Leica DFC320 color camera (Leica, Wetzlar, Germany).

### Immunofluorescence

Mouse brains at P5 and P10 were dissected and processed as previously described [22]. Specimens were paraffin embedded and cut into 8–10 μm sections, and IF staining carried out following standard protocols. Heat antigen retrieval (0.3% sodium citrate, 0.05%, Tween-20 pH 6.0) was performed followed by tissue permeabilization (0.5% Triton X-100 in PBS) for 15 minutes at RT. Slides were blocked for 1 hour at RT (5% goat/donkey serum, 1% BSA, 0.75% glycine, 0.5% Tween-20 in PBS), and incubated at 4°C O/N with primary antibody in a humidifying chamber. Primary antibodies against the following antigens were used: FOXJ1 (mouse, 1:500; 14–9965, eBiosciences, San Diego, CA), Glast (guinea pig, 1:1000; AB1783, EMD Millipore, Billerica, MA), Acα-Tub (mouse, 1:1000; T6793, Sigma-Aldrich, St. Louis, MO), Vimentin (rabbit, 1:500; ab92547, Abcam, Cambridge, MA), S100β (rabbit, 1:50; Z-0311, DAKO, Carpinteria, CA), N-cadherin (rabbit, 1:50; sc-7939, Santa Cruz Biotechnology, Santa Cruz, CA), β-catenin (mouse monoclonal, 1:250, 610153, BD Biosciences, San Jose, CA). The sections were incubated in secondary antibodies for 2 hours at RT: Alexa 488 goat anti-mouse (1:250, Thermo-Fisher, Waltham, MA), Cy3 goat anti-mouse (1:250, Jackson ImmunoResearch, West Grove, PA), Alexa 647 donkey anti-rabbit (1:250, Jackson ImmunoResearch, West Grove, PA), FITC goat anti-rabbit (1:250, Jackson ImmunoResearch, West Grove, PA). Slides were DAPI stained (D1306; Thermo-Fisher, Waltham, MA) and mounted using Vectashield mounting medium (Vector Laboratories, Burlingame, CA). Images were taken on a Zeiss Axiovert 200M deconvolution microscope equipped with Axiocam 105 color and 503 monochrome cameras (Zeiss, Göttingen, Germany).

### IF quantification

Quantification was carried out by examining slides in a blinded fashion, where an average of 5 animals per genotype and at least 3 sections per animal were prepared using numerical values only. Pictures were taken from comparable ventricular areas, with 2–3 fields per slide photographed randomly. Images were taken with the Zeiss Axiovert 200M deconvolution microscope, stored in tagged image file format and analyzed using FIJI ImageJ. At least 200 cells were counted in each experiment and the data represents the percent of cells examined in either ventral or dorsal ependyma. All images were taken with equivalent exposures across sections to allow for objective quantification. For the quantification of mislocalized proteins (i.e. N-cadherin and β-catenin), significant displacement throughout the basolateral membranes was required to score as mislocalized. Statistical analysis was performed with GraphPad Prism® 5.02 software (GraphPad Software, California, USA). Results are expressed as means ± standard deviation (SD). Statistical significance was calculated using a t-test and statistical significance was set to p< 0.05 (* p<0.05, ** p<0.01, *** p<0.001).

### Whole mount immunofluorescence

Medial and lateral ventricular walls from P10 mice were isolated in cold PBS and sectioned using a Leica VT1000S vibratome (Leica, Wetzlar, Germany). Tissue was fixed in 4% PFA in PBS O/N at 4°C, followed by blocking with 2.5% BSA, 0.2% Triton X-100 in PBS for 4 hours at RT. Primary antibodies used: N-cadherin (rabbit, 1:50; sc-7939, Santa Cruz Biotechnology, Santa Cruz, CA), γ-tubulin (mouse 1:250; ab11316, Abcam, Cambridge, MA). Samples were incubated with primary antibody for 24–48 hours at 4°C. Secondary antibody incubation was performed at 4°C for 48 hours using Alexa 488 goat anti-mouse (1:250, Thermo-Fisher, Waltham, MA) and Alexa 647 donkey anti-rabbit (1:250, Jackson ImmunoResearch, West Grove, PA). Tissues were placed on glass slides, mounted and imaged using an Andor WD Spinning Disk confocal microscope system equipped with an Andor Neo sCMOS camera (Andor, Belfast, UK).

### Scanning electron microscopy (SEM)

Brains collected at P10 were fixed in Karnovsky’s fixative (2.5% glutaraldehyde, 2% paraformaldehyde, 0.1 M sodium cacodylate buffer pH 7.4) for 7 days at 4°C. Brain ventricles were then split and fixed in Karnovsky’s for 4 additional days. Brains were washed in 0.1 M cacodylate buffer and dehydrated through series of ethanol washes. Dehydrated tissue was immersed in pure hexamethyldisilazane (HMDS) (EMS, Hatfield, PA) twice for 15 minutes at RT. Samples were covered in fresh HMDS and left under the hood until evaporated. Specimens were mounted and sputter coated with gold/palladium using a Polaron E5100 coater (Polaron Instruments, West Sussex, UK). Images were taken using a Hitachi S-3000N variable pressure scanning electron microscope (Hitachi, Tokyo, Japan).

### Rotational polarity analysis

Existing TEM micrographs of P5 ependymal cilia cross sections were reexamined to determine basal foot orientation. The right-most basal body in each image was randomly chosen to act as the reference. Using Fiji software (http://fiji.sc/Fiji), a line was drawn along the center of the basal body, terminating at the tip of the basal foot. This line was copied without varying the angle to each other basal body in the image. Independent lines were then drawn along the central length of each of the non-reference basal bodies in the cluster. If all basal feet were perfectly aligned, each pair of lines should be completely overlapping and the angle between them should be zero. If the lines were not fully overlapping, the Fiji angle tool was used to calculate the angle between them. To represent the full range of rotation from the reference, angles located to the right from a predominantly vertical reference line or above a predominantly horizontal reference line were labeled as positive, while those to the left or below the reference were labeled as negative.

### Recording and analysis of ependymal-generated flow

*In vivo* ciliary flow recordings were performed on P10 mouse ventricles. Lateral ventricles were isolated and immediately dissected in cold DMEM supplemented with 25 mM HEPES, followed by sectioning using a Leica VT1000S vibratome (Leica, Wetzlar, Germany) to a final thickness of 500 μm. Sections were maintained in DMEM containing 25 mM HEPES at RT during the recording period. FluoSpheres Carboxylate-Modified microspheres (0.5 μm yellow-green; F8813, Thermo-Fisher, Waltham, MA) were used to track the flow generated by ependymal cilia. Live images were recorded at 300 frames per second using an Ultima In Vivo multiphoton laser scanning microscope (Bruker Nano Fluorescence Microscopy, Billerica, MA) equipped with a 10X immersion objective and an optical zoom of 80X. Video rate image acquisition was achieved by using the Ultima system resonant scanners. Imaris software was used to analyze the tracked fluorescent particles and calculate the average speed of the beads (Bitplane AG, Zurich, Switzerland).

## Results

### Ventricular ependymal cells are morphologically abnormal in *Jhy*^*lacZ/lacZ*^ mice

Ependymal differentiation and motile ciliogenesis are coordinated, with cilia formation initiating as ependymal cells undergo morphological differentiation. Proper ependymal differentiation is likely required for ciliogenesis, as in mice lacking Foxj1, where the ependymal cells do not differentiate, they also do not undergo ciliogenesis. We considered that the morphological defects observed in the cilia of *Jhy*^*lacZ/lacZ*^ mice might be a result of altered ependymal cell differentiation, with changes in ciliogenesis secondary to a differentiation block. To assess the differentiation of *Jhy*^*lacZ/lacZ*^ radial glial cells to ependyma, we examined the expression patterns of cellular markers of undifferentiated radial glia and differentiated ependyma [7,8,35,36]. The gradient of normal ependymal differentiation means that the cells of the medial wall are noticeably advanced compared to cells of the lateral wall during the first postnatal week [6,15]. To be able to observe ependymal differentiation as early as possible during postnatal development, while brain deformation from the hydrocephalus remains limited, our studies were performed primarily on medial wall tissues from the lateral ventricles, the earliest-differentiating region. When it has been possible to analyze lateral wall tissues, i.e. once lateral ependymal differentiation has begun but before significant hydrocephalic changes occur, these data are presented as well.

**Fig 1:**
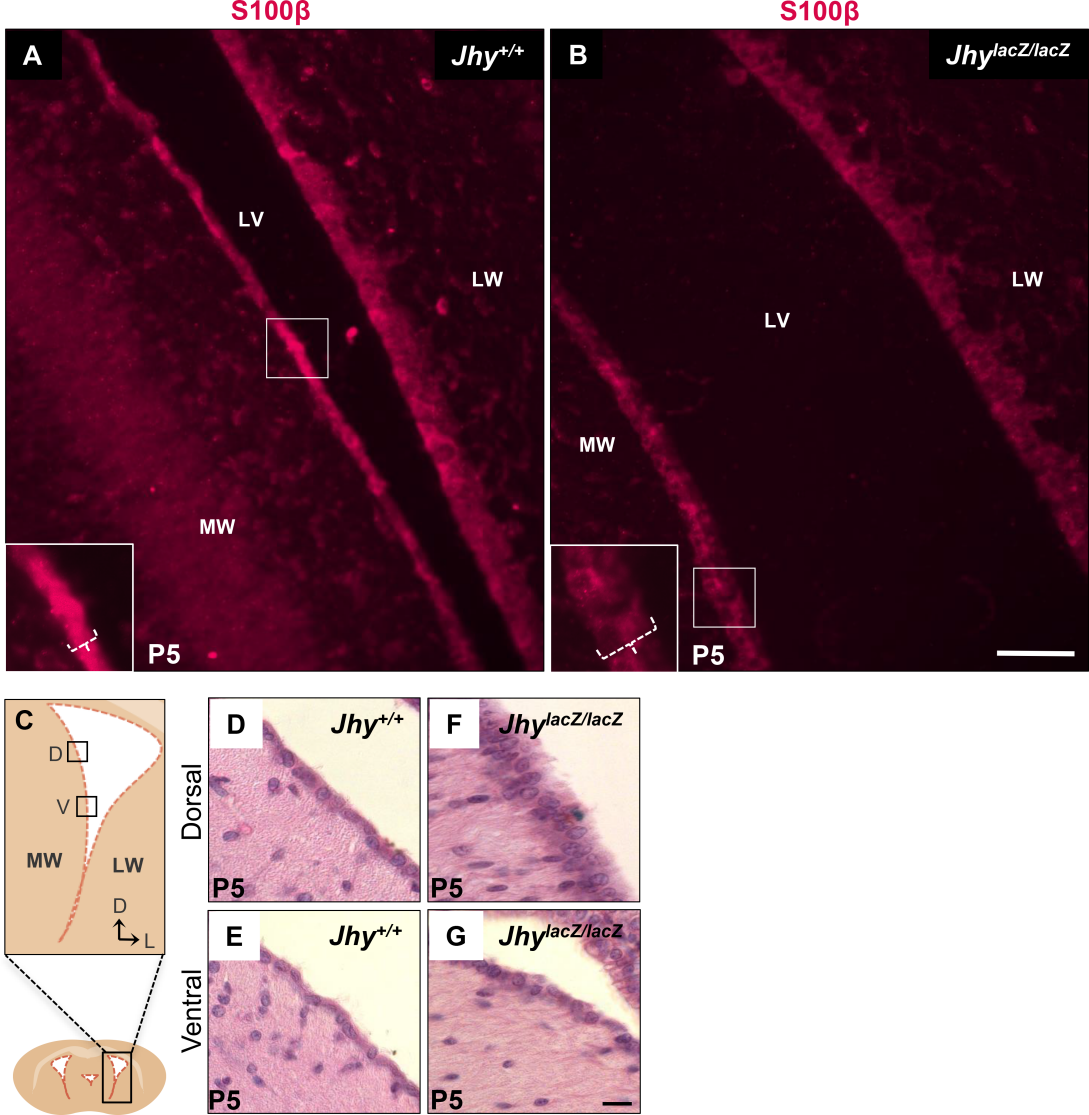
Delayed differentiation of medial ventricular wall ependymal cells in *Jhy*^*lacZ/lacZ*^ mice. (A, B) IF detection of S-100β in P5 coronal sections from *Jhy*^*+/+*^ (A) and *Jhy*^*lacZ/lacZ*^ (B) brains. The boxed region in each large image shows the region of the inset, and the white bracket denotes the width of the cell layers marked by S-100β expression. A) *Jhy*^*+/+*^ medial wall cells display a flattened, single cell-layered differentiated ependyma, while B) *Jhy*^*lacZ/lacZ*^ medial wall cells retain a pseudostratified undifferentiated appearance. (C) Schematic of lateral ventricle showing regions designated as medial wall and lateral wall, dorsal and ventral. (D-G) H&E staining of sections of P5 *Jhy*^*+/+*^ (D, E) and *Jhy*^*lacZ/lacZ*^ (F, G) lateral ventricle medial ependymal walls. *Jhy*^*+/+*^ ependyma has a differentiated appearance in both dorsal and ventral regions (D, E), while *Jhy*^*lacZ/lacZ*^ remains undifferentiated dorsally, but is largely differentiated ventrally (F, G). MW, medial wall; LW, lateral wall; LV, lateral ventricle; D, dorsal; V, ventral; L, lateral. Scale bars: 50μm (A-B); 20μm (D-G).

The ependymal cell marker S100β is an EF-hand calcium binding protein expressed in a variety of brain cell types, including all ventricular ependyma [37]. S100β protein was localized by immunofluorescence (IF) to examine the ependymal cell morphology of *Jhy*^*+/+*^ and *Jhy*^*lacZ/lacZ*^ animals as these cells undergo the differentiation process (Fig 1A and 1B). S100β expression in medial wall ependymal cells of *Jhy*^*+/+*^ mice at P5 highlights the flattened appearance characteristic of differentiated ependymal cells (Fig 1A, bracket in inset). In *Jhy*^*lacZ/lacZ*^ mice, however, medial wall ependymal cells retain a pseudostratified morphology, representative of undifferentiated radial glia (Fig 1B, bracket in inset). At this age, the later-differentiating lateral walls of both *Jhy*^*+/+*^ and *Jhy*^*lacZ/lacZ*^ ventricles show a pseudostratified layer of radial glial cells, indicating that differentiation has not yet begun in this region (Fig 1A and 1B). To more clearly examine ependymal cell morphology, histological analysis was performed on both the later differentiating dorsal, and earlier differentiating ventral, regions of the lateral ventricle at P5 (Fig 1C–1G). In *Jhy*^*+/+*^ brains, the medial wall displays a single layer of flattened ependymal cells both dorsally (Fig 1D) and ventrally (Fig 1E), indicating that morphological differentiation is complete by P5 in these animals. In *Jhy*^*lacZ/lacZ*^ brains, however, dorsal cells display a pseudostratified appearance resembling radial glia (Fig 1F), while ventral cells have taken on a more differentiated morphology (Fig 1G). These marked differences in differentiation were consistently observed in independent experiments using different animals (S1A–S1H Fig). These results suggest that ependymal cell differentiation is altered in the medial ventricular walls of *Jhy*^*lacZ/lacZ*^ mice, particularly in the dorsal region. As expected at P5, the lateral wall cells appear undifferentiated by histological analysis in both dorsal and ventral regions of both *Jhy*^*+/+*^ and *Jhy*^*lacZ/lacZ*^, similar to what was seen with S100β (S2A–S2D Fig).

### *Jhy*^*lacZ/lacZ*^ mice show delayed ependymal cell differentiation

**Fig 2:**
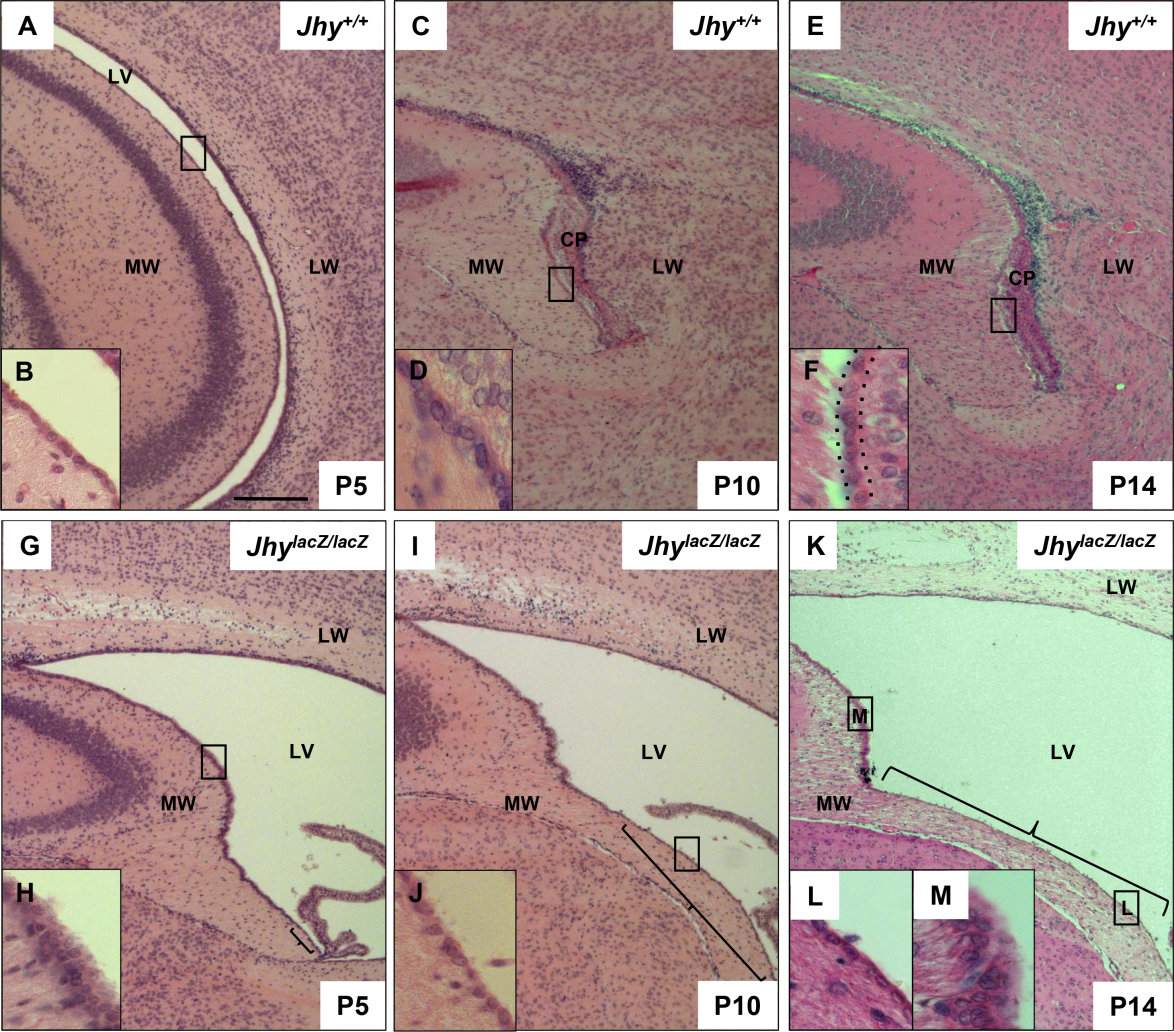
Ventromedial ependymal cells progressively acquire differentiated ependymal characteristics. H&E staining of coronal sections of P5 (A, G), P10 (C, I) and P14 (E, K) *Jhy*^*+/+*^ and *Jhy*^*lacZ/lacZ*^ lateral ventricles. *Jhy*^*+/+*^ medial wall ependymal cells at P5 (A, inset B), P10 (C, inset D) and P14 (E, inset F) display differentiated characteristics in both dorsal and ventral regions. The boxed region in each large image shows the area of the inset, the dotted line in F indicates the medial ependymal cell layer. *Jhy*^*lacZ/lacZ*^ dorsal cells remain undifferentiated at P5 (G, inset H), P10 (I), and P14 (K, inset M). *Jhy*^*lacZ/lacZ*^ ventral cells progressively acquire a differentiated appearance (inset J, L), with the differentiated (bracketed) region advancing dorsally (G, I, K). CP, choroid plexus; MW, medial wall; LW, lateral wall; LV, lateral ventricle. Scale bars: 100μm (A-M).

To better track the delay in ependymal differentiation in *Jhy*^*lacZ/lacZ*^ mice, a time course of histology was performed. Sections were prepared from *Jhy*^*+/+*^ and *Jhy*^*lacZ/lacZ*^ animals at P5, P10 and P14, and examined for medial wall ependymal cell differentiation. It was not possible to extend the time course beyond P14, as the overall morphology of *Jhy*^*lacZ/lacZ*^ brains became significantly altered by the worsening hydrocephalus. As expected, *Jhy*^*+/+*^ brains display a monolayer of differentiated ependymal cells both dorsally and ventrally by P5, which persists at P10 and P14 (Fig 2A, 2C and 2E, insets 2B, 2D and 2F). (Note the choroid plexus tissue that lies between the closely opposed medial and lateral walls of *Jhy*^*+/+*^ mice at P10 and P14, which floats freely in the enlarged ventricle in *Jhy*^*lacZ/lacZ*^.) In *Jhy*^*lacZ/lacZ*^ mice, a small region of the ventral-most cells appear to be differentiated at P5 (Fig 2G, bracket), but the cells are increasingly undifferentiated in more dorsal regions (inset H). At P10, the region of differentiated cells has lengthened dorsally (Fig 2I, bracket, inset 2J), moving as a wave-like transition towards the roof of the ventricle. At P14, the differentiated ventral region has further lengthened (Fig 2K, bracket, inset 2L), but the most dorsal cells remain undifferentiated at all ages that could be examined (Fig 2K, inset 2M). *Jhy*^*lacZ/lacZ*^ ependymal cell differentiation is therefore delayed at least 9 days beyond the time point at which the last *Jhy*^*+/+*^ dorsal cells are differentiated. These results indicate that *Jhy*^*lacZ/lacZ*^ medial wall ependymal cells do continue to differentiate in the expected pattern, although we were unable to assay if the process is ever fully completed. Lateral wall ependymal cells, in both *Jhy*^*+/+*^ and *Jhy*^*lacZ/lacZ*^ mice, appear largely differentiated at P10, at least by morphological criteria.

### *Jhy*^*lacZ/lacZ*^ ependymal cells retain expression of a radial glial marker

**Fig 3:**
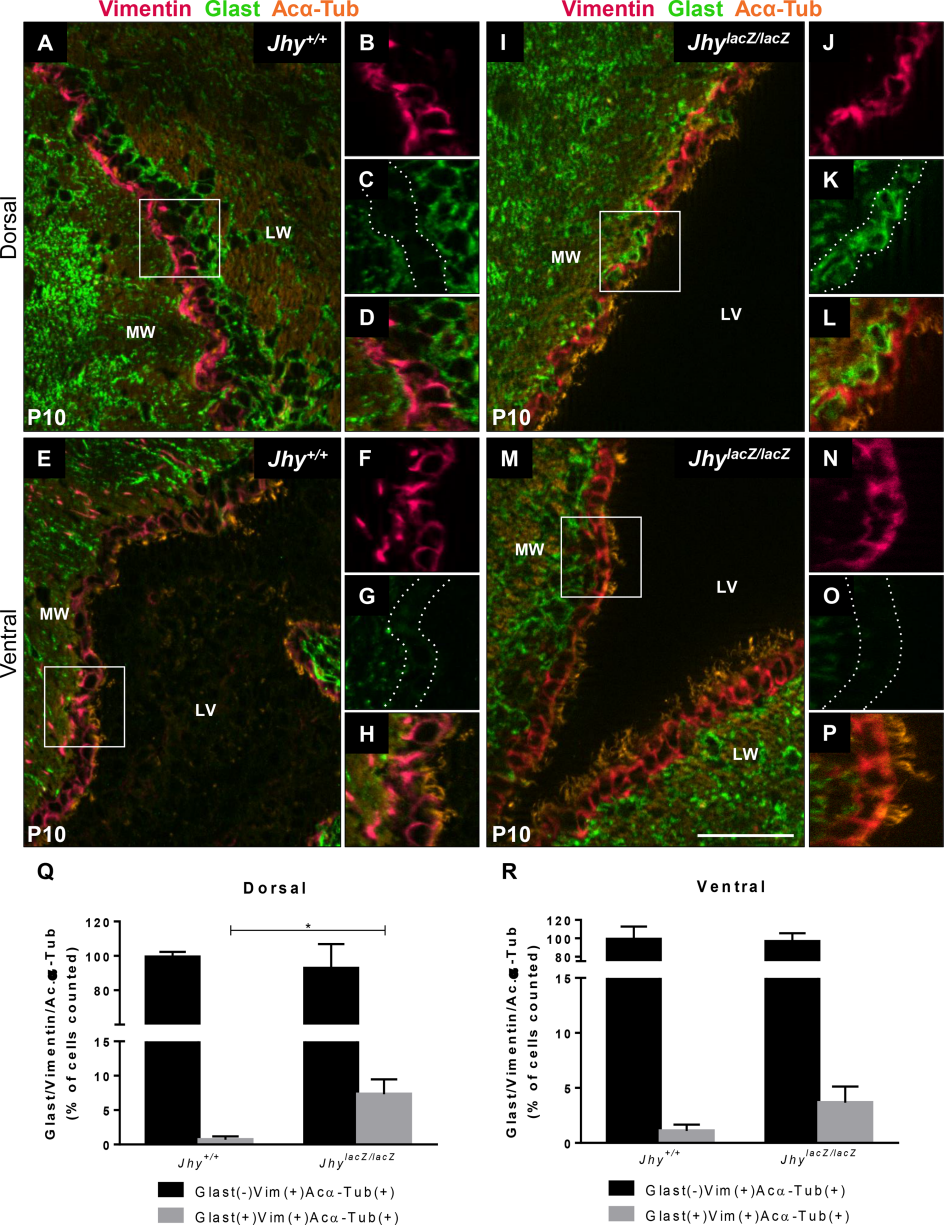
*Jhy*^*lacZ/lacZ*^ mice exhibit delayed radial glial to ependymal cell differentiation. IF analysis of P10 lateral ventricle coronal sections from *Jhy*^*+/+*^ (A, E) and *Jhy*^*lacZ/lacZ*^ (I, M) mice for expression of Vimentin (pink), Glast (green) and Acα-Tub (orange) in dorsal (A-D, I-L) and ventral (E-H, M-P) brain regions. Lower right panels (D, L, H, P) represent a higher magnification view of the merged image. In *Jhy*^*+/+*^, medial wall dorsal and ventral cells express the differentiated ependymal markers Vimentin (A, B, E, F) and Acα-Tub (A, D, E, H), but are negative for the radial glial marker Glast (A, C, E, G). In *Jhy*^*lacZ/lacZ*^ brains, some dorsal cells remain positive for the undifferentiated marker Glast (I, K), while also expressing the differentiated markers Vimentin and Acα-Tub (I, J, L). *Jhy*^*lacZ/lacZ*^ ventral cells express only Vimentin and Acα-Tub (M-P). The dotted line indicates the medial wall ependymal cells in (C, G, K, O). (Q-R) Graphical representation of the percentage of Glast(-)Vimentin(+)Acα-Tub(+) (black bar) and Glast(+)Vimentin(+)Acα-Tub(+) (grey bar) cells in dorsal (Q) and ventral (R) ependymal cells. MW, medial wall; LW, lateral wall; LV, lateral ventricle; * denotes p≤0.05. Scale bars: 50μm (A-P).

The delay in radial glial to ependymal differentiation was further characterized by IF using stage-specific markers. Glast is a glutamate transporter that is highly expressed in radial glial cells, but downregulated upon ependymal differentiation [6,8,38]. The intermediate filament protein Vimentin is a marker of differentiated ependymal cells, and acetylated α-Tubulin (Acα-Tub) can be used to visualize the ependymal cilia once they emerge [8,10]. These markers were used to analyze *Jhy*^*+/+*^ (Fig 3A and 3E) and *Jhy*^*lacZ/lacZ*^ (Fig 3I and 3M) medial ventricular wall at P10. Quantification of visible gene expression in differentiated and undifferentiated ependymal cells was performed on *Jhy*^*+/+*^ and *Jhy*^*lacZ/lacZ*^ brains (Fig 3Q and 3R). *Jhy*^*+/+*^ dorsal (Fig 3A–3D and 3Q) and ventral (Fig 3E–3H and 3R) cells are Glast(-)Vimentin(+)Acα-Tub(+), indicative of fully differentiated ependymal cells. In *Jhy*^*lacZ/lacZ*^ mice, however, some dorsal cells retain expression of the radial glial cell marker Glast, while also expressing the ependymal-specific markers Vimentin and Acα-Tub (Glast(+)Vimentin(+)Acα-Tub(+), compare C to K and D to L), a pattern not seen in wild type cells (Fig 3I–3L and 3Q). These cells appear to be neither undifferentiated radial glia, nor differentiated ependyma, but stalled at an intermediate stage that *Jhy*^*+/+*^ cells rapidly transit. *Jhy*^*lacZ/lacZ*^ ventral cells are Glast(-)Vimentin(+)Acα-Tub(+), confirming the histological data that shows these cells are differentiated (Fig 3M–3P and 3R). These data indicate that ventral cells, which appear morphologically differentiated at P10, are also differentiated on the basis of their gene expression (Fig 3M–3P). Dorsal cells, however, which remain morphologically undifferentiated, retain the expression of the undifferentiated marker Glast (Fig 3I–3L). These Glast(+)Vimentin(+)Acα-Tub(+) cells account for a small, but statistically significant, percentage of dorsal ependyma in *Jhy*^*lacZ/lacZ*^ (Fig 3Q). Nevertheless, the Glast(+) regions of the *Jhy*^*lacZ/lacZ*^ medial wall ependyma appeared to be too few in number to account for the near complete failure of differentiation by morphological criteria. The failure to silence Glast is therefore unlikely to be the primary cause of the differentiation delay, but rather one consequence of a broader defect in ependymal differentiation, and further examination of ependymal markers is warranted. We also analyzed the expression of differentiation markers in the later-differentiating lateral ependymal wall at P5. Lateral wall of both *Jhy*^*+/+*^ and *Jhy*^*lacZ/lacZ*^ mice have ependymal cells that are Glast(-)Vimentin(+), in both dorsal and ventral regions (S2E–S2H Fig). At this developmental stage it appears that the lateral walls in *Jhy*^*+/+*^ and *Jhy*^*lacZ/lacZ*^ mice are equivalent in their differentiation status based on gene expression criteria.

### FOXJ1 expression is unaltered in *Jhy*^*lacZ/lacZ*^ mice

**Fig 4:**
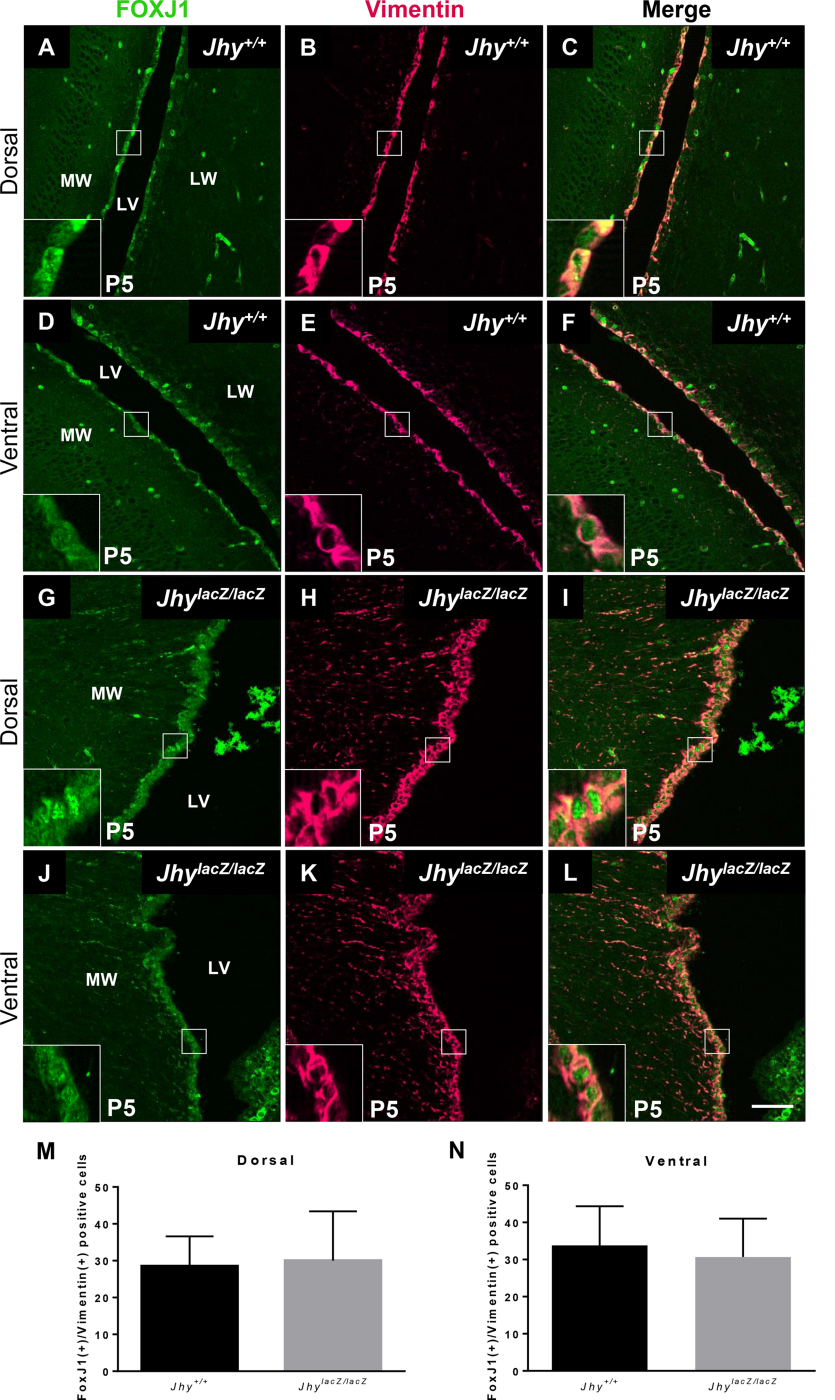
Delayed *Jhy*^*lacZ/lacZ*^ ependymal cells express FOXJ1 normally. IF analysis of P5 lateral ventricle medial wall sections from *Jhy*^*+/+*^ (A-F) and *Jhy*^*lacZ/lacZ*^ (G-L) mice for expression of Vimentin (pink) and FOXJ1 (green). *Jhy*^*+/+*^ dorsal (A-C) and ventral (D-F) cells express the differentiated ependymal markers Vimentin and FOXJ1. In *Jhy*^*lacZ/lacZ*^ brains, both undifferentiated-appearing dorsal (G-I) and differentiated-appearing ventral (J-L) cells express Vimentin and FOXJ1. (M-N) Quantification of the percentage of cells positive for FoxJ1 dorsally (M) and ventrally (N) in *Jhy*^*+/+*^ (black bar) and *Jhy*^*lacZ/lacZ*^ (grey bar) mice. The boxed region in each large image shows the region of the inset. CP, choroid plexus; MW, medial wall; LW, lateral wall; LV, lateral ventricle. Scale bars: 50μm (A-L).

The transcription factor FOXJ1 is upregulated as radial glia differentiate to ependymal cells, and is required for proper ependymal development and ciliogenesis [39]. Altered expression of FOXJ1 might underlie the defects in *Jhy*^*lacZ/lacZ*^ ependymal differentiation, with the retention of Glast expression a consequence of an altered developmental program. To determine if FOXJ1 is involved in the *Jhy*^*lacZ/lacZ*^ ependymal defects, FOXJ1 expression was examined by IF in P5 lateral ventricle medial wall (Fig 4). In *Jhy*^*+/+*^ mice, ependymal cells are positive for FOXJ1 in both dorsal (Fig 4A–4C) and ventral (Fig 4D–4F) regions. Vimentin was used as a marker for differentiated ependymal cells, and colocalizes with FOXJ1 in all cells. *Jhy*^*lacZ/lacZ*^ ependymal cells are also positive for FOXJ1, in both undifferentiated-appearing dorsal (Fig 4G–4I), and differentiated-appearing ventral (Fig 4J–4L), regions of the medial wall. There was no significant difference in the number of FoxJ1 positive cells in both dorsal (Fig 4M) and ventral (Fig 4N) ependymal cells of *Jhy*^*lacZ/lacZ*^ brains. These results indicate that while P5 *Jhy*^*lacZ/lacZ*^ ependymal cells retain an undifferentiated morphology, and continue to express a radial glial marker, they do activate the ependymal differentiation protein FOXJ1. The cause of the differentiation delay in *Jhy*^*lacZ/lacZ*^ ependyma appears to lie downstream of FOXJ1, but upstream of Glast.

### N-cadherin is mislocalized from *Jhy*^*lacZ/lacZ*^ adherens junctions

The integrity of the radial glial cell layer, and the later ependyma, is maintained by junctional complexes that bind cells at their adjacent membranes, and tether these membranes to the internal cytoskeleton [40–42]. Adherens junctions (AJs) are one form of junctional complex, and in addition to their structural roles AJs function as signaling centers through interactions with cellular proteins such as the α- and β-catenins [43–45]. The AJ complex is defined by the presence of one of several cadherins, transmembrane proteins that interact with the homologous domains of cadherins on adjacent cells, and N-cadherin is the primary AJ protein expressed in the developing brain [14,46]. The N-cadherin dimer is bound intracellularly by P120 and β-catenin, and then by α-catenin, which links the complex to the actin filaments of the cytoskeleton, either alone or through the protein Vinculin [40]. AJs are strictly required in ependymal cells, and alterations in the expression or localization of cadherins, or other AJ-associated proteins, cause disruptions in the ependymal cell layer [14].

**Fig 5:**
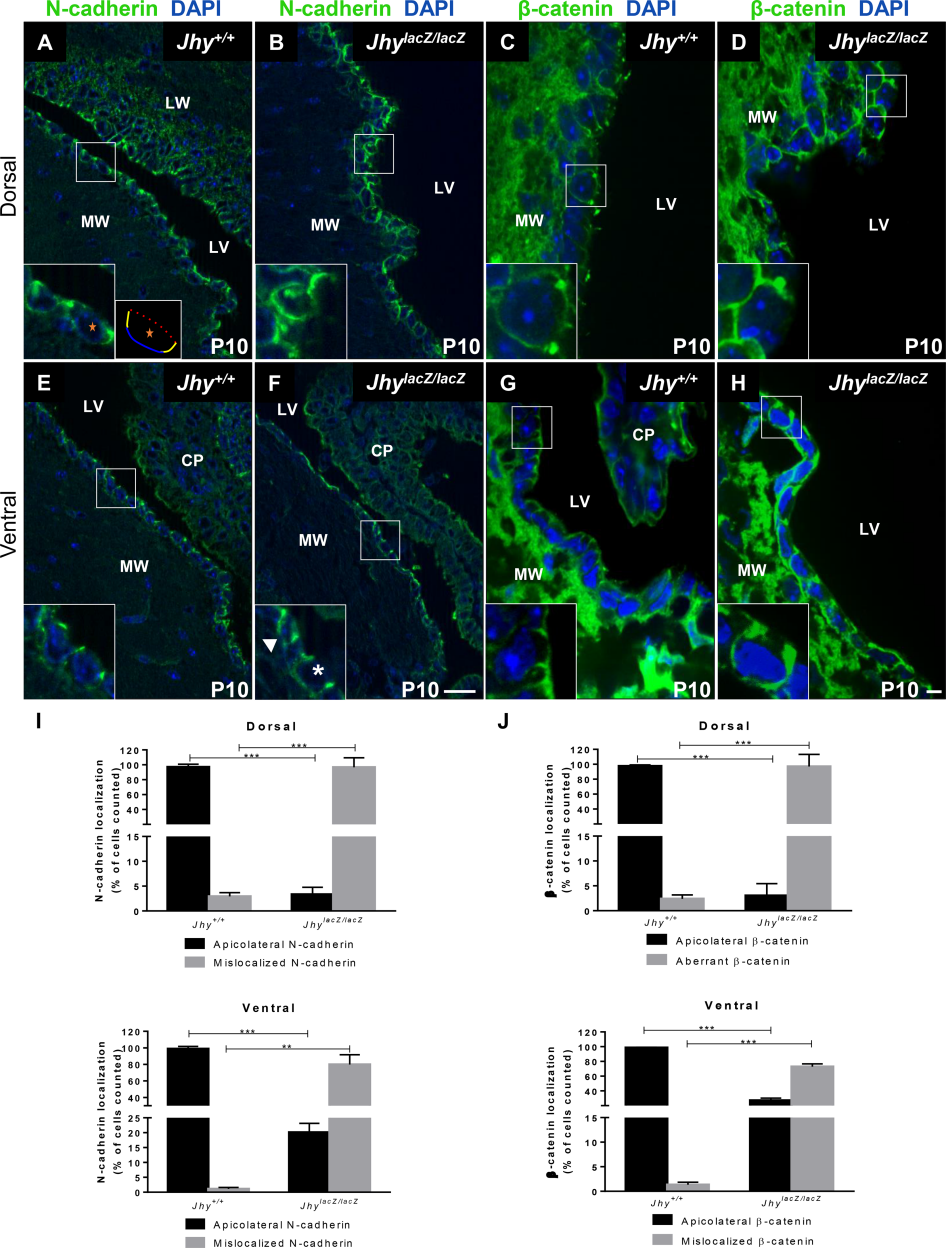
Abnormal N-cadherin and β-catenin localization in *Jhy*^*lacZ/lacZ*^ ependymal cells. In P10 *Jhy*^*+/+*^ medial wall ependymal cells, N-cadherin (A, E) and β-catenin (C, G) expression in dorsal (A, C) and ventral (E, G) regions localize to the adherens junctions at the apicolateral cell borders (inset in A, C). A schematic of a typical cell in panel A (marked by an orange star) is provided for orientation. In this schematic, the apical cell membrane is indicated by a dotted red line, the basal membrane by a blue line, and the lateral membranes by yellow lines. In *Jhy*^*lacZ/lacZ*^ ependyma, dorsal cells showed mislocalization of N-cadherin and β-catenin (B, D), with both proteins found throughout the basolateral cell membrane (inset in B, D). Some *Jhy*^*lacZ/lacZ*^ dorsal cells also contained large cytoplasmic inclusions that were positive for N-cadherin (inset in B). Some *Jhy*^*lacZ/lacZ*^ ventral cells display proper apicolateral N-cadherin localization (inset in F, asterisk), while other cells mislocalize N-cadherin throughout the lateral walls (inset in F, arrowhead). All ventral cells in *Jhy*^*lacZ/lacZ*^ showed mislocalization of β-catenin throughout the basolateral membranes (H). (I-J) Quantification of the percentage of cells displaying normal (black bar) and aberrant (grey bar) localization of N-cadherin (I) and β–catenin (J) in *Jhy*^*+/+*^ and *Jhy*^*lacZ/lacZ*^ medial wall ependymal cells. **, p≤0.01; ***, p≤0.001. DAPI is depicted in blue, the boxed region in each large image shows the region of the inset. CP, choroid plexus; MW, medial wall; LW, lateral wall; LV, lateral ventricle. Scale bars: 20μm bar in F (A, B, E, F); 10μm bar in H (C, D, G, H).

We investigated the structural integrity of AJs in *Jhy*^*lacZ/lacZ*^ mice by localization of their components. In *Jhy*^*+/+*^ dorsal and ventral ependymal cells at P10, N-cadherin expression is localized to the most apical part of the lateral cell membranes, referred to as the apicolateral cell border, which is the site of the AJs (Fig 5A and 5E). In *Jhy*^*lacZ/lacZ*^ dorsal cells, however, which are morphologically undifferentiated, N-cadherin localization is disrupted, with protein found throughout the basal and lateral membranes (Fig 5B). In cells with N-cadherin mislocalization, this protein is also retained intracellularly, in large aggregates or inclusions (Fig 5B). N-cadherin localization is disrupted in the differentiated-appearing ventral *Jhy*^*lacZ/lacZ*^ cells as well, though less significantly so, with smaller amounts of basolateral signal and more protein localizing to AJs (Fig 5F and 5I). Quantification of N-cadherin localization showed a significant decrease in apicolateral protein, and an increase in basal/lateral protein, in both dorsal and ventral *Jhy*^*lacZ/lacZ*^ cells as compared to *Jhy*^*+/+*^ (Fig 5I). N-cadherin mislocalization was not restricted to the medial ventricular wall, as basolateral accumulation of the protein is also found in the lateral wall of *Jhy*^*lacZ/lacZ*^ ventricles at P10 (S2I–S2J Fig). These data indicate that the ventromedial *Jhy*^*lacZ/lacZ*^ cells, which are delayed but do eventually appear morphologically differentiated, are still abnormal by other parameters. The same appears to be true for the lateral wall ependyma of *Jhy*^*lacZ/lacZ*^ animals.

Cadherin levels at the AJs are regulated by balancing membrane transport with endocytosis, recycling and degradation, and these structures undergo dynamic reorganization as cells differentiate [47]. We asked whether the N-cadherin mislocalization of *Jhy*^*lacZ/lacZ*^ animals was first apparent in differentiating ependymal cells, or if it precedes this event. Radial glial cells also carry AJs, and these were analyzed in *Jhy*^*+/+*^ and *Jhy*^*lacZ/lacZ*^ mice at P0.5, the onset of ependymal differentiation. All radial glial cells, in *Jhy*^*+/+*^ ventricle and in both dorsal and ventral regions of the *Jhy*^*lacZ/lacZ*^ medial wall, show proper apicolateral localization of N-cadherin (S3 Fig). This data provides a temporal reference for the onset of N-cadherin mislocalization, suggesting the impairment arises with the beginning of ependymal differentiation.

### β-catenin localization to AJs is also disrupted in *Jhy*^*lacZ/lacZ*^ mice

The mislocalization of N-cadherin in *Jhy*^*lacZ/lacZ*^ ependyma prompted us to examine the distribution of β-catenin, a core AJ component that mediates adhesion as well as signaling [48,49]. β-catenin is an effector of the WNT pathway, but cytoplasmic β-catenin is usually ubiquitinated and degraded. Activation of WNT signaling stabilizes β-catenin, allowing it to translocate to the nucleus where it functions in transcriptional activation [50,51]. β-catenin binding to N-cadherin sequesters it at the AJ, preventing both its degradation and its nuclear translocation. In *Jhy*^*+/+*^ ependyma at P10, β-catenin is concentrated at the apicolateral AJs, with only minor signal at the basolateral membranes (Fig 5C and 5G). In *Jhy*^*lacZ/lacZ*^ ependyma, β-catenin is still found at the AJs, but increased protein is also present throughout the basolateral membrane (Fig 5D and 5H). Quantification showed that the number of cells with aberrant localization of β-catenin was significantly increased in *Jhy*^*lacZ/lacZ*^ medial wall ependymal cells when compared to *Jhy*^*+/+*^ mice (Fig 5J). The disruption of *Jhy*^*lacZ/lacZ*^ AJ components is therefore not limited to N-cadherin localization, but involves the β-catenin signaling molecule as well.

### Dorsal and ventral ependymal motile cilia are abnormal in *Jhy*^*lacZ/lacZ*^ mice

**Fig 6:**
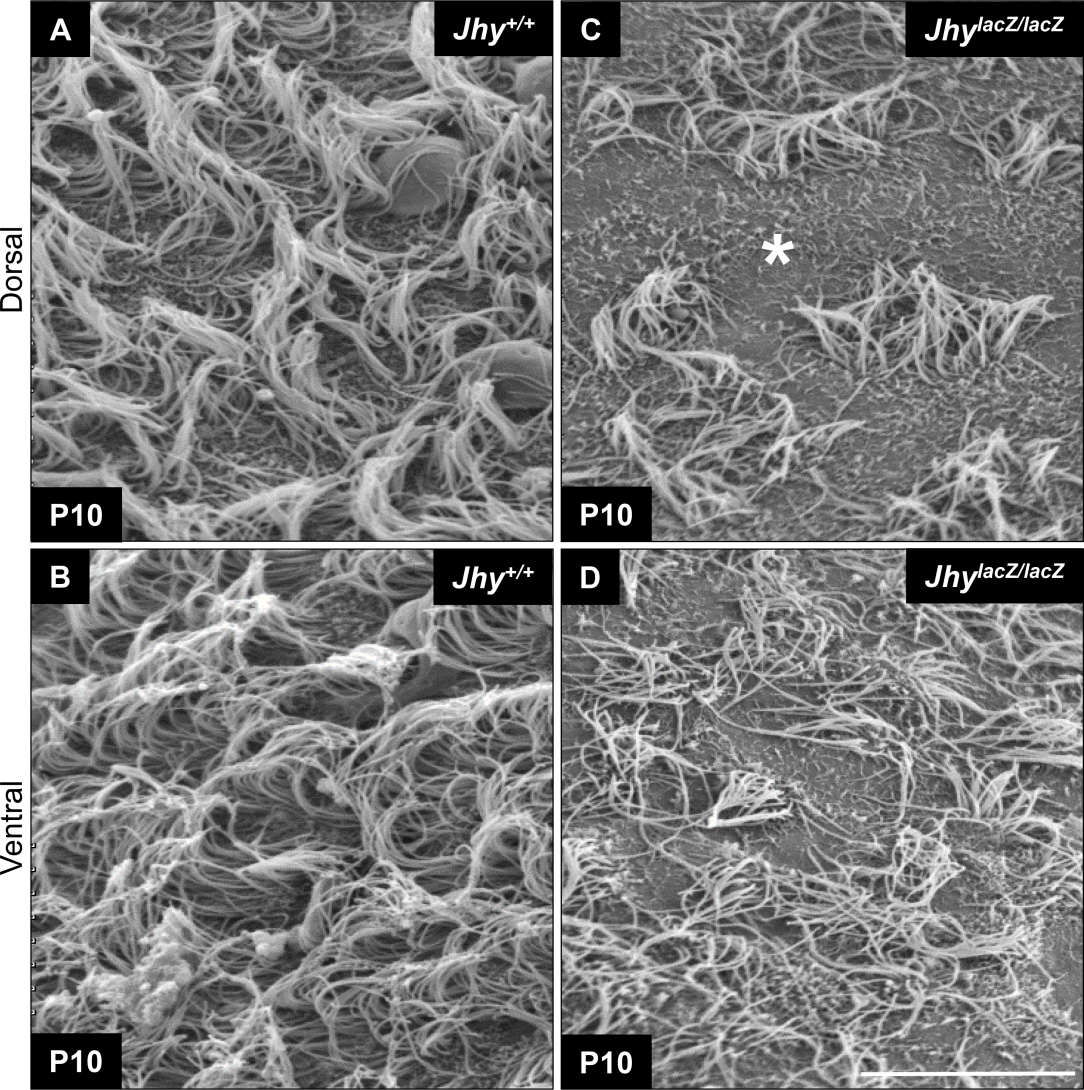
Ciliary abnormalities are more severe in the delayed *Jhy*^*lacZ/lacZ*^ dorsal ependymal cells. Scanning electron microscopy of lateral ventricle medial wall at P10 in *Jhy*^*+/+*^ (A, B) and *Jhy*^*lacZ/lacZ*^ (C, D). *Jhy*^*+/+*^ dorsal (A) and ventral (B) ependymal cells carry apical tufts of elongated cilia with a consistent orientation. In *Jhy*^*lacZ/lacZ*^ mice, both dorsal (C) and ventral (D) cells have fewer, disorganized cilia, with areas where cells have no cilia at all (C, asterisk). Dorsal cells were typically more severely affected than were ventral cells in *Jhy*^*lacZ/lacZ*^ brains. Scale bars: 20μm (A-D).

Delayed ependymal cell differentiation could underlie the abnormal ciliogenesis of *Jhy*^*lacZ/lacZ*^ animals [22]. As the onset of ciliogenesis is poorly understood, it may be that if the delayed *Jhy*^*lacZ/lacZ*^ ependymal cells do not initiate ciliogenesis within a certain developmental window, proper ciliary morphogenesis and patterning may be impossible. In this case, we might expect the ciliary defects to be more severe in the dorsal region of the ventricle, where the differentiation delay is most pronounced, and less so in the ventral region. Our previous electron microscopy studies did not compare different regions of the ventricle, so scanning electron microscopy (SEM) was carried out focusing specifically on dorsal and ventral regions of the medial wall. In P10 *Jhy*^*+/+*^mice, both dorsal and ventral ependymal cells carry abundant long cilia with a consistent directional orientation (Fig 6A and 6B). The cilia of *Jhy*^*lacZ/lacZ*^ mice are less abundant than in *Jhy*^*+/+*^, as we have shown previously, and individual ciliary bundles are disorganized and randomly oriented (Fig 6C and 6D). While both dorsal and ventral *Jhy*^*lacZ/lacZ*^ cilia are abnormal, extensive imaging suggests a more severe effect in dorsal cells that have the greatest differentiation delay. Specifically, dorsal ependyma has a greater percentage of cells that carry no cilia at all (Fig 6C). These may be the Glast-expressing undifferentiated ependymal cells that were identified by IF, which are most severely affected and never progress to ciliogenesis.

### Impaired ciliary-generated flow in *Jhy*^*lacZ/lacZ*^ mice

**Fig 7:**
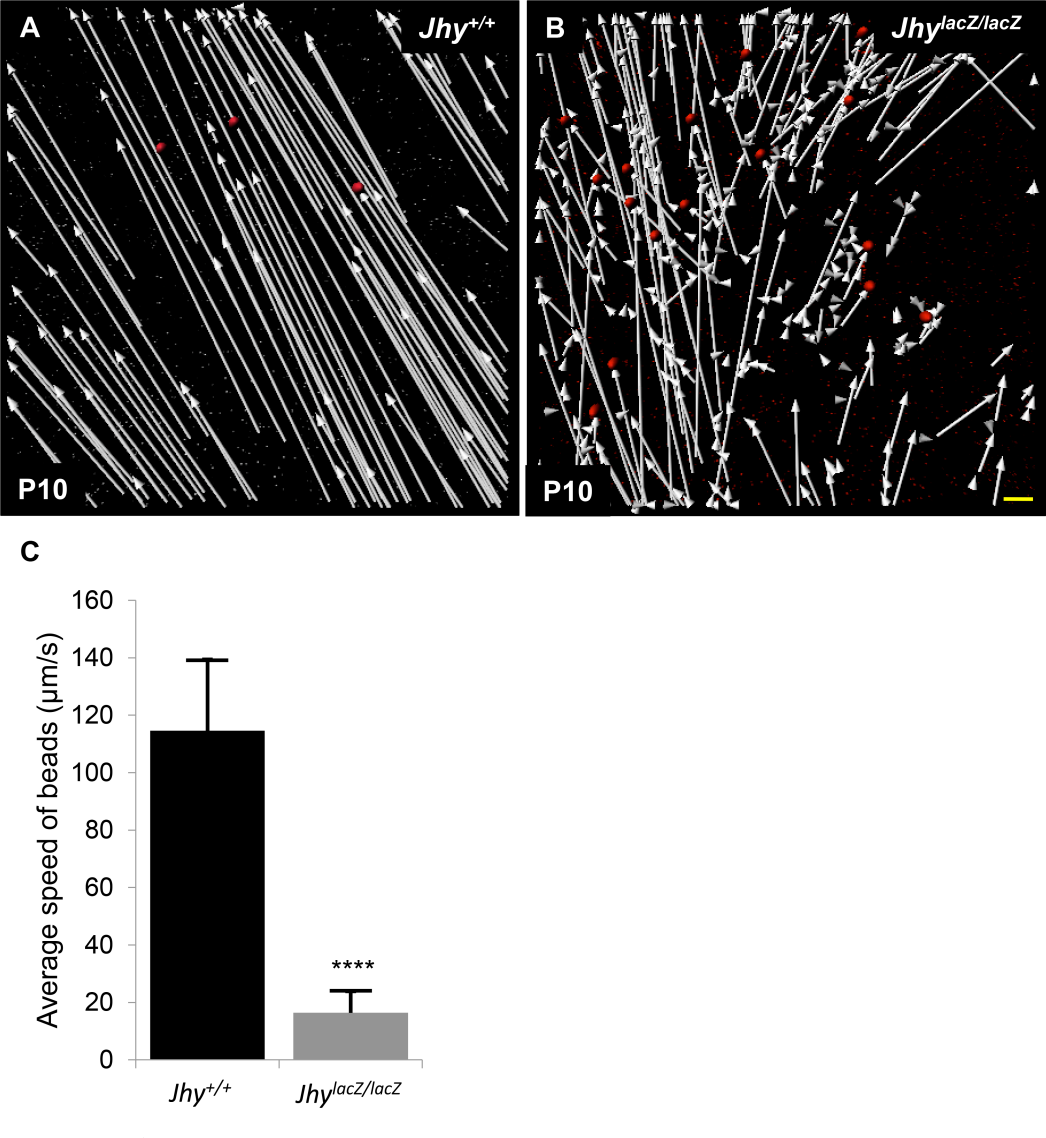
Impaired cilia-generated flow in *Jhy*^*lacZ/lacZ*^ mice. (A, B) High-speed video imaging of fluorescent bead movement on *Jhy*^*+/+*^ (A) and *Jhy*^*lacZ/lacZ*^ (B) ventricular wall explants to measure speed and directionality of ciliary flow. Vectors indicating bead tracks from *Jhy*^*+/+*^ mice were long and strongly directional (A), while vectors from *Jhy*^*lacZ/lacZ*^ mice were much shorter with disorganized movement and poor directionality (B). (C) Graphical representation of average bead speed in *Jhy*^*+/+*^ (black bar) and *Jhy*^*lacZ/lacZ*^ (grey bar) explants shows the greatly reduced flow generated by *Jhy*^*lacZ/lacZ*^ cilia. Red circles represent fluorescent beads; **** denotes p≤0.0001. Scale bars: 15μm (A-B).

Previously we suggested that the morphologically abnormal ependymal cilia of *Jhy*^*lacZ/lacZ*^ mice, and particularly the loss of the central microtubule pair, mean that these cilia will not exhibit normal movement, but this had not been proven. To examine the flow produced by *Jhy*^*lacZ/lacZ*^ cilia, we performed high-speed video imaging of ventricular wall sections. Ventricular tissues were removed from *Jhy*^*+/+*^ and *Jhy*^*lacZ/lacZ*^ P10 animals, and ependymal vibratome sections quickly prepared. Fluorescent microbeads were applied to one end of the section under microscopic imaging and bead speed and directionality were recorded. In *Jhy*^*+/+*^ samples, beads moved quickly across the ventricular surface, with strong linear vectors and an average speed of 114 μm/second (Fig 7A and 7C, S1 Video). *Jhy*^*lacZ/lacZ*^ cilia were able to generate only minimal flow, with beads displaying irregular short tracks, and moving at speeds averaging 16 μm/second (Fig 7B and 7C, S2 Video). The morphologically abnormal *Jhy*^*lacZ/lacZ*^ cilia therefore cannot generate significant fluid flow, likely causing or contributing to the hydrocephalus of *Jhy*^*lacZ/lacZ*^ mice.

### Compromised translational and rotational polarity in *Jhy*^*lacZ/lacZ*^ ependyma

The noncanonical WNT/planar cell polarity (PCP) signaling pathway directs the unified polarization of epithelial cells across a tissue surface [52]. PCP pathway factors localize asymmetrically within individual cells, and then act to translate that asymmetry into a reorganization of cell polarity. PCP mechanisms underlie numerous events during embryonic development, including the orientation of the motile cilia of the ependyma [53]. During ciliogenesis, PCP controls the asymmetric clustering of the basal bodies to one end of each ependymal cell (translational polarity), the maintenance of this polarity across a tissue surface (tissue polarity), and the unidirectional orientation of individual basal bodies within each ciliary bundle (rotational polarity) [54–56].

**Fig 8:**
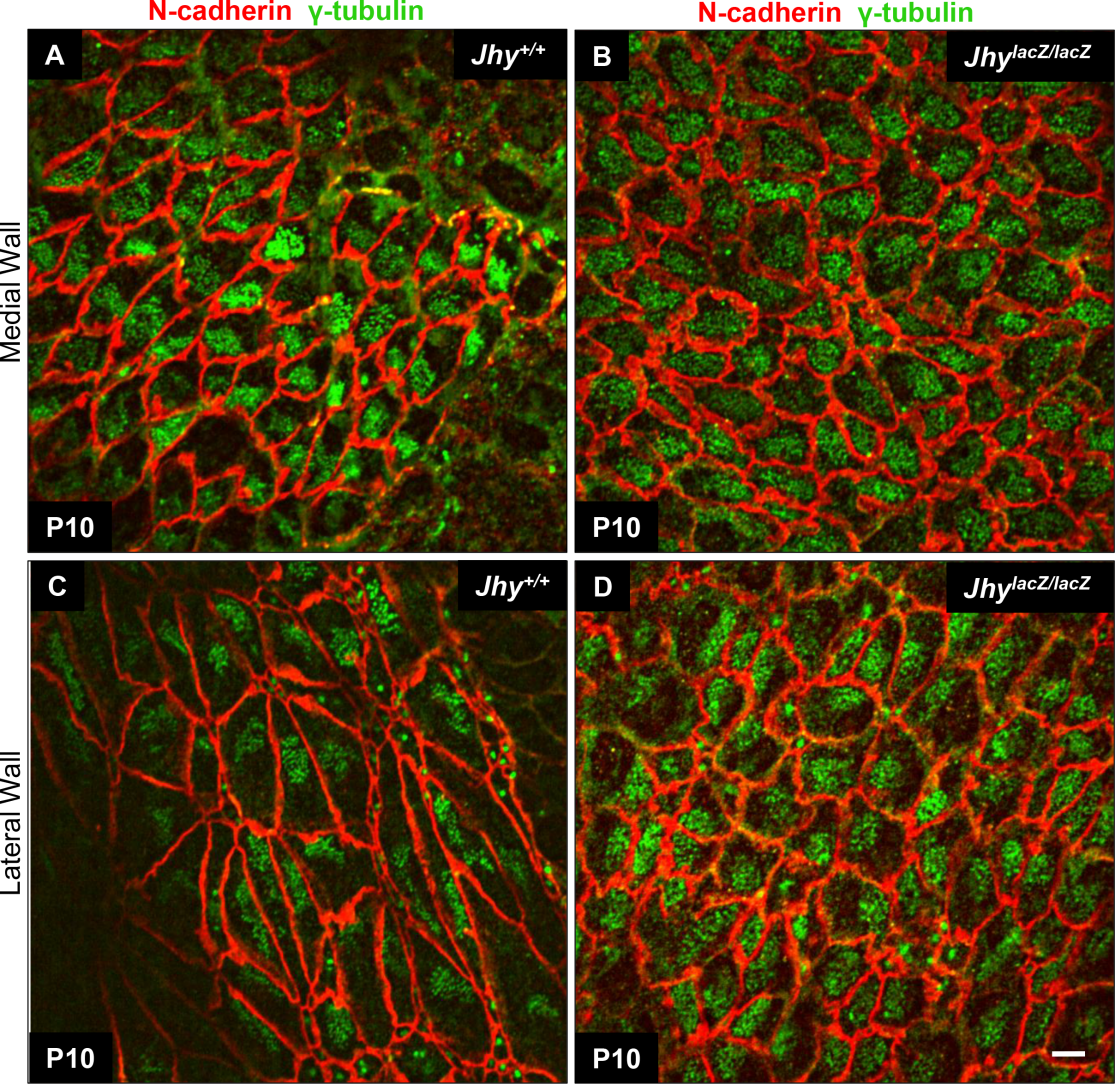
Translational polarity is disrupted in *Jhy*^*lacZ/lacZ*^ ependymal cells. Whole mount IF of P10 medial (A, B) and lateral (C, D) wall of lateral ventricle ependyma. N-cadherin (red) delineates the cell boundaries, while γ-tubulin (green) marks basal body patches on the apical cell surface. Medial wall ependymal cells in *Jhy*^*+/+*^ display basal body polarization to one end of the cell, while lateral wall basal bodies are only partly polarized at this stage (A, C). In *Jhy*^*lacZ/lacZ*^ medial wall, basal body patches are located centrally within the cells, and display little evidence of polarization (B). In *Jhy*^*lacZ/lacZ*^ lateral walls, some cells display a degree of polarization, while others have centrally located basal bodies (D). Both the medial and lateral walls of *Jhy*^*lacZ/lacZ*^ ependyma display morphological changes as well, with thickened cell membranes that may reflect the altered distribution of N-cadherin (B, D). *Jhy*^*lacZ/lacZ*^ lateral wall cells are also much smaller and rounder in shape than their *Jhy*^*+/+*^ counterparts (D). Scale bars: 10μm (A-D).

To evaluate the translational polarity of *Jhy*^*lacZ/lacZ*^ ependymal cilia, whole mount IF was performed on P10 ventricular walls. An antibody against γ-tubulin was used to label the ciliary basal bodies, and an antibody against N-cadherin was used to highlight the cell membranes. *Jhy*^*+/+*^ medial and lateral wall tissues show the presence of elongated ependymal cells expressing abundant N-cadherin at their lateral borders (Fig 8A and 8C). Although ciliogenesis is not complete at P10, medial wall ependymal cells of *Jhy*^*+/+*^ mice already display an asymmetrical clustering of basal bodies towards one end of most cells, and this was largely maintained across the ependymal surface (Fig 8A). Lateral wall cells are larger in size than medial wall cells, and display the typical “rosette” appearance of ependymal cells organized around a central neural stem cell (Fig 8C) [57]. These cells have inconsistent basal body polarization, perhaps reflecting the earlier stage of their differentiation process (Fig 8C). In *Jhy*^*lacZ/lacZ*^ tissues, both medial and lateral wall ependymal cells have membranes that appeared thickened, with a more irregular shape than those of *Jhy*^*+/+*^ cells (Fig 8B and 8D). This likely reflects the broadened distribution of N-cadherin protein already described (Fig 5, S2 Fig). *Jhy*^*lacZ/lacZ*^ lateral wall cells also appear smaller in size and rounder in shape than the elongated cells seen in *Jhy*^*+/+*^ lateral walls, and show fewer rosette structures (Fig 8C and 8D). In *Jhy*^*lacZ/lacZ*^ medial and lateral walls, γ-tubulin showed that the basal bodies are distributed across the apical cell surface with little polarization (Fig 8B and 8D). This loss of translational polarity means that tissue polarity, an extension of translational polarity, cannot be assessed.

**Fig 9:**
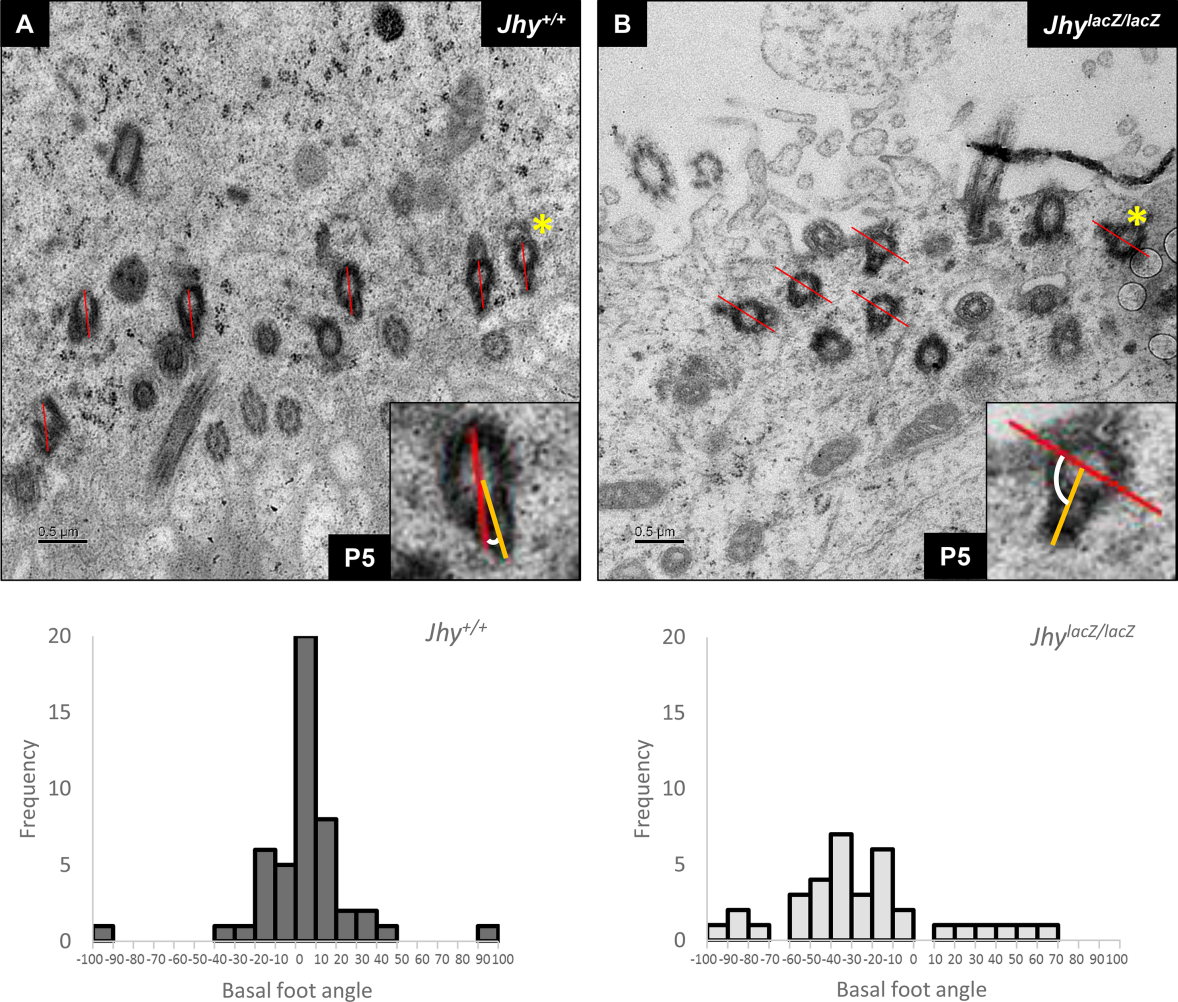
Impaired rotational polarity in *Jhy*^*lacZ/lacZ*^ mice. (A, B) TEM images of P5 ependyma from *Jhy*^*+/+*^ (A) and *Jhy*^*lacZ/lacZ*^ (B) mice. The rightmost basal foot was chosen as the reference (white asterisk) in each image, and a line (red) was drawn from the outer edge of the basal body through the center of the basal foot. This line was copied unchanged to other basal bodies in the cluster, then a second line (yellow) was drawn indicating the actual orientation of each basal foot, and the angle of offset between each line pair was calculated. By way of example, the insets in panels A and B show the angle calculation for the basal foot that is boxed in the larger panel. Even at P5, rotational polarity is well established in *Jhy*^*+/+*^ (A), with only small angles of deviation (C). In *Jhy*^*lacZ/lacZ*^, however, basal foot alignment is far more variable (B, D). (C, D) Histogram showing the rotational angles of basal feet in *Jhy*^*+/+*^ (C) (n = 68) and *Jhy*^*lacZ/lacZ*^ (D) ependyma (n = 49). Scale bars: 0.5μm (A-B).

To investigate the maintenance of rotational polarity in *Jhy*^*lacZ/lacZ*^ ependymal cells, lateral ventricle TEM sections generated previously were reexamined for orientation of the ciliary basal feet [22]. The basal foot is an anchoring structure found at the base of each cilia, which determines the direction of ciliary motion. Basal foot rotation reflects the orientation of the microtubule central pair, also an indicator of the direction of motility, but exactly how these two structures are linked is unknown. During early ciliogenesis, cilia arise without rotational polarity, and the basal feet point in random directions. As the planar cell polarity pathway begins to organize each ciliary cluster, individual basal bodies rotate to align all basal feet within the cluster. Although the brains examined in these experiments were P5, younger than the tissues examined for translational polarity, *Jhy*^*+/+*^ animals already showed significant polarization of the basal feet (Fig 9A and 9C). Within each cilia cluster, the majority of basal feet were aligned along a single plane (Fig 9A), and the angle of deviation from a randomly selected reference basal foot was small (Fig 9C). In equivalent sections of *Jhy*^*lacZ/lacZ*^ mice, however, basal feet remained randomly positioned (Fig 9B), with a far greater range of deviation across a single cluster (Fig 9D). These data indicate a disruption of the PCP signals controlling multiple aspects of cell polarity in *Jhy*^*lacZ/lacZ*^ ependymal cells.

## Discussion

Mice lacking the JHY protein product of the *Jhy* gene (*Jhy*^*lacZ/lacZ*^) develop juvenile hydrocephalus as early as postnatal day 1.5, and rarely survive past 6 weeks of age [22]. We showed previously that these mice have sparser and shorter ependymal motile cilia, and most lack the central pair of microtubules that is required for ciliary motility. We have now determined that these abnormal cilia are nearly immotile, and incapable of generating significant fluid flow, the likely cause of *Jhy*^*lacZ/lacZ*^ hydrocephalus (Fig 7 and S1 and S2 Videos). We show that in addition to controlling the generation, patterning and organization of motile cilia, *Jhy* plays a larger role in regulating ependymal cell differentiation in the mouse. The *Jhy* gene is also implicated in the development and function of cilia in other species, and is under the control of the ciliogenesis program initiated by FOXJ1 and other factors. The human *Jhy* ortholog (*c11orf63*) was identified in a proteomic study of ciliated tissues, with expression in epithelia of the lung and oviduct [58]. Choksi et al found that the zebrafish *Jhy* ortholog (*C10h11orf63*) is upregulated in transgenic zebrafish overexpressing the ciliogenesis regulator *FoxJ1* [59].

The ciliary defects of *Jhy*^*lacZ/lacZ*^ mice led us to more closely examine ependymal cells during the postnatal differentiation period. We find that *Jhy*^*lacZ/lacZ*^ medial wall ependymal cells are delayed in their differentiation by at least 9 days when compared to *Jhy*^*+/+*^ mice (Fig 2). *Jhy*^*+/+*^ cells have acquired the monolayer typical of differentiated ependyma by P5, while *Jhy*^*lacZ/lacZ*^ cells are not fully differentiated even by P14. This phenotype is most severe in the dorsal regions of the lateral ventricles, which are the last cells to differentiate in wild type animals. Some *Jhy*^*lacZ/lacZ*^ ependymal cells also retain expression of the radial glial marker Glast, beyond the time at which this gene is silenced in *Jhy*^*+/+*^ cells (Fig 3). *Jhy*^*lacZ/lacZ*^ cells do express the ependymal marker FOXJ1, however, indicating that they are capable of initiating the differentiation program. The coexpression of Glast and FOXJ1 in some *Jhy*^*lacZ/lacZ*^ cells suggests they are stalled in a transitional state between undifferentiated radial glia and differentiated ependyma, a stage quickly transitioned in wild type ependymal cells. Detailed SEM imaging showed that while both ventrally- and dorsally-located ependymal cells make abnormal cilia in *Jhy*^*lacZ/lacZ*^ mice, the more significantly delayed dorsal cells more often lack cilia entirely (Fig 6C). These may represent the cells that coexpress Glast/Vim/Acα-Tub, and remain stalled in the differentiation process. How are delayed ependymal differentiation and abnormal ciliogenesis correlated? For a process that is highly temporally regulated, we speculate that there may be a window in which differentiating ependymal cells are competent for ciliogenesis. Beyond such time, the activators and/or components required for ciliogenesis may be unavailable. The increasing differentiation delay from ventral to dorsal ependyma might therefore cause a progressive defect in ciliary number and morphology.

*Jhy*^*lacZ/lacZ*^ ependymal cells show altered localization of the adherens junction proteins N-cadherin and β-catenin, with reduced protein at the apicolateral adherens junctions, and more distributed throughout the basolateral membranes (Fig 5B and 5F). The undifferentiated dorsal cells often have intracellular inclusions of N-cadherin, suggesting a defect in the process of AJ remodeling that accompanies many developmental transitions. The pattern seen in *Jhy*^*lacZ/lacZ*^ ependymal cells could result from reduced N-cadherin transport to the apicolateral AJs, increased removal from the AJs, and/or an inability recycle the protein. The presence of normal N-cadherin localization in *Jhy*^*lacZ/lacZ*^ radial glial cells at P0.5 indicates a defect that begins only with the onset of ependymal differentiation (S3 Fig). Despite the AJ alterations, *Jhy*^*lacZ/lacZ*^ ependymal cells remain a discrete epithelial monolayer and do not detach from the underlying tissue, i.e. there is no ependymal denudation as is seen in some models of cadherin depletion [14,60–62]. The *Jhy*^*lacZ/lacZ*^ phenotype therefore manifests less severely than a complete loss of cadherin function.

In addition to their structural role, AJs have signaling functions that are mediated through interactions with a diverse array of proteins [44,63,64]. N-cadherin binding of β-catenin retains this protein at the AJs, preventing its translocation and downregulating the WNT response [65]. AJ localization of β-catenin also prevents its degradation, providing a pool of inactive but quickly releasable protein if WNT signaling is activated. N-cadherin and β-catenin do not first associate at the AJ, but form a complex in the cytoplasm prior to their transport to the membrane [66]. It is not surprising then that β-catenin is also mislocalized in *Jhy*^*lacZ/lacZ*^ ependymal cells (Fig 5D and 5H). Altered recycling and/or redelivery of AJ components to the plasma membrane may underlie the mislocalization of both N-cadherin and β-catenin.

The direction of motile cilia bending is determined by the orientation of the basal bodies that organize each cilium, and the positioning of the central microtubule pair. The translational and rotational polarity of the basal bodies are under the control of the WNT/PCP pathway, which shares components with canonical WNT signaling [53,55,67]. *Jhy*^*lacZ/lacZ*^ animals show a loss of translational polarity of the ependymal cilia, with basal bodies remaining dispersed across the apical cell surface (Fig 8B and 8D). The lack of a consistent basal foot orientation in *Jhy*^*lacZ/lacZ*^ ciliary bundles indicates loss of rotational polarity in these animals as well, which may be correlated with the missing central pairs (Fig 9B and 9D). There is precedent for defects in PCP signaling altering ependymal cilia number, morphology and polarity, and these defects often result in hydrocephalus in animal models [10,54,56].

In this and earlier work we have shown that inactivation of *Jhy* causes defects in 1) ependymal cell differentiation, 2) ciliary length and number, 3) microtubule organization, 4) polarity of ependymal cilia, and 5) N-cadherin transport and AJ establishment. What function(s) might *Jhy* have that its loss causes such varied effects? We propose that most aspects of the *Jhy*^*lacZ/lacZ*^ phenotype can be attributed to the failure to establish N-cadherin complexes at adherens junctions in differentiating ependymal cells. AJs contribute structure and tensile strength to an epithelial cell layer, and constrain the shape and movement of cells through organization of the cytoskeleton [68]. Remodeling of AJs accompanies morphological changes during development, including those of the neuroepithelium as it differentiates to radial glia, and then to neurons and ependyma [68–70]. Defects in AJ structure in *Jhy*^*lacZ/lacZ*^ mice may affect the ability of radial glia to transform from cuboidal cells to the flattened ependymal shape, and/or to undergo the movements necessary to refine into a single cell layer. This would be reflected as altered tissue morphology in histological cross-sections, and altered cell shape in whole mount analyses, as we have demonstrated.

The signaling functions of AJs intersect numerous cell differentiation cascades, including the WNT, Epidermal Growth Factor (EGF), and TGF-β pathways, and changes in AJ structure have been found to alter developmental signaling [71,72]. N-cadherin recruitment of β-catenin to the plasma membrane decreases the signaling-competent pool of this transactivator, and cells with reduced levels of N-cadherin show increased LEF-mediated transcriptional activation through β-catenin [73,74]. Changes in N-cadherin/β-catenin localization at *Jhy*^*lacZ/lacZ*^ AJs could function through two possible mechanisms to affect WNT signaling pathways. Reduction in the levels of N-cadherin at AJs might result in less β-catenin recruited to these sites, with a concomitant increase in signaling-competent β-catenin in the cytoplasm that can potentiate WNT-driven effects. Alternatively, ectopically localized N-cadherin in the basolateral membranes could titrate activated β-catenin and reduce its nuclear function. To our knowledge canonical WNT signaling thru β-catenin has not been characterized in ependymal cells, so the functions of this pathway, or the potential ligands involved, are unknown. Discriminating between these possibilities could be accomplished by the construction of a WNT-responsive signaling system in ependymal cells, in which JHY and N-cadherin levels can be manipulated.

Lastly, altered interactions between cadherins and components of the PCP pathway may underlie the defects in polarity seen in *Jhy*^*lacZ/lacZ*^ ependymal cells. The PCP pathway has been suggested to control the cytoskeletal reorganization that regulates basal body positioning and ciliogenesis during ependymal cell differentiation [10,56]. For example, loss of Celsr2, a PCP component, perturbs the actin cytoskeleton network, impairing proper basal body arrangement [54]. Recent work has shown direct interactions between cell adhesion molecules and PCP proteins, for example the core PCP protein Vangl2 binds to N-cadherin in developing neurons [75]. Both deletion and overexpression of Vangl2 cause adhesion defects in these cells, with altered distribution of N-cadherin and β-catenin, suggesting dosage of Vangl2/N-cadherin is critical [76]. There is cross-talk between the canonical (β-catenin) and noncanonical (PCP) WNT pathways that converge at the AJ. β-catenin and Vangl2 bind to the same domain of N-cadherin, and these interactions are mutually exclusive. β-catenin binding stabilizes N-cadherin at the membrane, while Vangl2 binding promotes its removal, so these factors may compete to control N-cadherin localization and abundance [75]. We propose that the JHY protein acts during ependymal cell differentiation to accomplish N-cadherin targeting to, or stabilization at, the apicolateral membrane. Loss of this function not only upsets the distribution of N-cadherin, but also impacts the many developmental signaling pathways with AJ components.

It is less clear how loss of JHY results in the lack of the ciliary microtubule central pair; particularly since the factors that position the central pair are not known. It is possible that this defect is a consequence of the overall delay in ciliary development. Alternatively, it may be that the PCP pathway has an unacknowledged role in microtubule positioning that is affected in *Jhy*^*lacZ/lacZ*^ ependyma. Basal foot rotation parallels the orientation of the central pair [67], suggesting coordinated processes may position these two structures. The loss of rotational polarity and the central pair in *Jhy*^*lacZ/lacZ*^ animals may reflect a broader inability to polarize individual cilia in the absence of *Jhy*. Lastly, although not explored in this work, we cannot rule out a role for defects in primary cilia structure and/or function being involved in the *Jhy*^*lacZ*^ phenotype.

## Supporting information

S1 Fig Loss of *Jhy* expression in *Jhy*^*lacZ/lacZ*^ mice delays ependymal cell differentiation. H&E staining of P5 medial wall ependyma in *Jhy*^*+/+*^ (A-D) and *Jhy*^*lacZ/lacZ*^ (E-H). Dorsomedial (A, C) and ventromedial (B, D) ependyma in *Jhy*^*+/+*^ display a flattened differentiated appearance. Similar morphological characteristics are observed in ventral ependymal cells in *Jhy*^*lacZ/lacZ*^ (F, H), yet undifferentiated ependymal cells are still observed in dorsally located regions (E, G). Scale bars: 20μm (A-H).
(TIF)

S2 Fig Altered lateral wall ependymal cell morphology, with aberrant N- cadherin localization, in *Jhy*^*lacZ/lacZ*^ mice. H&E staining of P5 lateral walls in both *Jhy*^*+/+*^ (A, C) and *Jhy*^*lacZ/lacZ*^ (B, D) animals show undifferentiated cuboidal ependyma in both dorsal (A, B) and ventral (C, D) regions of the lateral wall. Lateral wall sections were used for IF for Vimentin (pink) and Glast (green) from *Jhy*^*+/+*^ (E, G) and *Jhy*^*lacZ/lacZ*^ (F, H) brains. In both *Jhy*^*+/+*^ (E, G) and *Jhy*^*lacZ/lacZ*^ (F, H), both dorsal (E, F) and ventral (G, H) cells were Glast(-)Vimentin(+). N-cadherin IF (green) in P10 brain shows normal apicolateral localization in *Jhy*^*+/+*^ (I, inset), while *Jhy*^*lacZ/lacZ*^ lateral wall ependyma display abnormal basolateral N-cadherin localization (J, inset). CP, choroid plexus; MW, medial wall; LW, lateral wall; LV, lateral ventricle. Scale bars: 50μm (A-D); 20μm (E-H); 20μm (I-J).
(TIF)

S3 Fig *Jhy*^*lacZ/lacZ*^ radial glia progenitors show normal N-cadherin localization. N-cadherin (green) IF in P0.5 medial wall of *Jhy*^*+/+*^ (A, C) and *Jhy*^*lacZ/lacZ*^ (B, D). *Jhy*^*+/+*^ dorsal (A) and ventral (C) ependyma display normal apicolateral N-cadherin localization. *Jhy*^*lacZ/lacZ*^ dorsal (B) and ventral (D) ependyma also show N-cadherin localized to the expected apicolateral position. CP, choroid plexus; MW, medial wall; LW, lateral wall; LV, lateral ventricle. Scale bars: 50μm (A-D).
(TIF)

S1 Video High-speed video imaging of fluorescent bead movement on *Jhy*^*+/+*^ ventricular wall explants to measure speed and directionality of ciliary flow. *Jhy*^*+/+*^ cilia produced rapid and highly directional movement of the labeled beads across the ventricular surface.
(MP4)

S2 Video High-speed video imaging of fluorescent bead movement on *Jhy*^*lacZ/lacZ*^ ventricular wall explants to measure speed and directionality of ciliary flow. *Jhy*^*lacZ/lacZ*^ cilia produced minimal bead movement, i.e. minimal flow, with no consistent directionality.
(MP4)

## References

[1] Garcia-Verdugo J, Ferron S, Flames N, Collado L, Desfilis E, Font E. The proliferative ventricular zone in adult vertebrates: a comparative study using reptiles, birds, and mammals. Brain Res Bull. 2002;57: 765–775. 12031273 10.1016/s0361-9230(01)00769-9

[2] Bruni J. Ependymal development, proliferation, and functions: a review. Microscopy Res Tech. 1998;41: 2–13. 10.1002/(SICI)1097-0029(19980401)41:1<2::AID-JEMT2>3.0.CO;2-Z 9550133

[3] Bruni J, Del Bigio M, Clattenburg R. Ependyma: normal and pathological. A review of the literature. Brain Res. 1985;356: 1–19. 3888350 10.1016/0165-0173(85)90016-5

[4] Del Bigio M. Ependymal cells: biology and pathology. Acta Neuropathol. 2010;119: 55–73. doi: 10.1007/s00401-009-0624-y 20024659 10.1007/s00401-009-0624-y

[5] Coskun V, Wu H, Blanchi B, Tsao S, Kim K, Zhao J, Biancotti J, Hutnick L, Krueger R Jr, Fan G G, de Vellis J, Sun Y. CD133+ neural stem cells in the ependyma of mammalian postnatal forebrain. Proc Natl Acad Sci USA. 2008;105: 1026–1031. doi: 10.1073/pnas.0710000105 18195354 10.1073/pnas.0710000105 PMC2242680

[6] Kuo C, Mirzadeh Z, Soriano-Navarro M, Rasin M, Wang D, Shen J, Sestan N, Garcia-Verdugo J, Alvarez-Buylla A, Jan L, Jan Y. Postnatal deletion of Numb/Numblike reveals repair and remodeling capacity in the subventricular neurogenic niche. Cell. 2006;127: 1253–1264. doi: 10.1016/j.cell.2006.10.041 17174898 10.1016/j.cell.2006.10.041 PMC1876765

[7] Jacquet B, Muthusamy N, Sommerville L, Xiao G, Liang H, Zhang Y, Holtzman M, Ghashghaei H. Specification of a Foxj1-dependent lineage in the forebrain is required for embryonic-to-postnatal transition of neurogenesis in the olfactory bulb. J Neurosci. 2011;31: 9368–9382. doi: 10.1523/JNEUROSCI.0171-11.2011 21697387 10.1523/JNEUROSCI.0171-11.2011 PMC3145804

[8] Lavado A, Oliver G. Six3 is required for ependymal cell maturation. Development. 2011;138: 5291–5300. doi: 10.1242/dev.067470 22071110 10.1242/dev.067470 PMC3222208

[9] Peng X, Lin Q, Liu Y, Jin Y, Druso J, Antonyak M, Guan J, Cerione R. Inactivation of Cdc42 in embryonic brain results in hydrocephalus with ependymal cell defects in mice. Protein Cell. 2013;4: 231–242. doi: 10.1007/s13238-012-2098-2 23150167 10.1007/s13238-012-2098-2 PMC3632363

[10] Tissir F, Qu Y, Montcouquiol M, Zhou L, Komatsu K, Shi D, Fujimori T, Labeau J, Tyteca D, Courtoy P, Poumay Y, Uemura T, Goffinet A. Lack of cadherins Celsr2 and Celsr3 impairs ependymal ciliogenesis, leading to fatal hydrocephalus. Nat Neurosci. 2010;13: 700–707. doi: 10.1038/nn.2555 20473291 10.1038/nn.2555

[11] Lechtreck KF, Delmotte P, Robinson ML, Sanderson MJ, Witman GB. Mutations in Hydin impair ciliary motility in mice. J Cell Biol. 2008;180: 633–643. doi: 10.1083/jcb.200710162 18250199 10.1083/jcb.200710162 PMC2234243

[12] Lechtreck KF, Witman GB. Chlamydomonas reinhardtii hydin is a central pair protein required for flagellar motility. J Cell Biol. 2007;176: 473–482. doi: 10.1083/jcb.200611115 17296796 10.1083/jcb.200611115 PMC2063982

[13] Davy BE, Robinson ML. Congenital hydrocephalus in hy3 mice is caused by a frameshift mutation in Hydin, a large novel gene. Hum Mol Genet. 2003;12: 1163–1170. 12719380 10.1093/hmg/ddg122

[14] Rodriguez E, Guerra M, Vio K, Gonzalez C, Ortloff A, Batiz L, Rodriguez S, Jara M, Munoz R, Ortega E, Jaque J, Guerra F, Sival D, den Dunnen W, Jimenez A, Dominguez-Pinos M, Perez-Figares JM, McAllister J, Johanson C. A cell junction pathology of neural stem cells leads to abnormal neurogenesis and hydrocephalus. Biol Res. 2012;45: 231–242. doi: 10.4067/S0716-97602012000300005 23283433 10.4067/S0716-97602012000300005

[15] Spassky N, Merkle FT, Flames N, Tramontin AD, Garcia-Verdugo JM, Alvarez-Buylla A. Adult ependymal cells are postmitotic and are derived from radial glial cells during embryogenesis. J Neurosci. 2005;25: 10–18. doi: 10.1523/JNEUROSCI.1108-04.2005 15634762 10.1523/JNEUROSCI.1108-04.2005 PMC6725217

[16] Tramontin A, Garcia-Verdugo J, Lim D, Alvarez-Buylla A. Postnatal development of radial glia and the ventricular zone (VZ): a continuum of the neural stem cell compartment. Cereb Cortex. 2003;13: 580–587. 12764031 10.1093/cercor/13.6.580

[17] Ashburner M, Ball C, Blake J, Botstein D, Butler H, Cherry J, Davis AP, Dolinski K, Dwight S, Eppig J, Harris M, Hill D, Issel-Tarver L, Kasarskis A, Lewis S, Matese J, Richardson J, Ringwald M, Rubin GM, Sherlock G. Gene ontology: tool for the unification of biology. Nat Genet. 2000;25: 25–29. doi: 10.1038/75556 10802651 10.1038/75556 PMC3037419

[18] Brody S, Yan X, Wuerffel M, Song S, Shapiro S. Ciliogenesis and left-right axis defects in forkhead factor HFH-4-null mice. Am J Respir Cell Mol Biol. 2000;23: 45–51. doi: 10.1165/ajrcmb.23.1.4070 10873152 10.1165/ajrcmb.23.1.4070

[19] Jacquet BV, Salinas-Mondragon R, Liang H, Therit B, Buie JD, Dykstra M, Campbell K, Ostrowski LE, Brody SL, Ghashghaei HT. FoxJ1-dependent gene expression is required for differentiation of radial glia into ependymal cells and a subset of astrocytes in the postnatal brain. Development. 2009;136: 4021–4031. doi: 10.1242/dev.041129 19906869 10.1242/dev.041129 PMC3118431

[20] Stubbs J, Oishi I, Izpisua Belmonte J, Kintner C. The forkhead protein Foxj1 specifies node-like cilia in Xenopus and zebrafish embryos. Nat Genet. 2008;40: 1454–1460. doi: 10.1038/ng.267 19011629 10.1038/ng.267 PMC4648715

[21] O'Callaghan C, Sikand K, Chilvers M. Analysis of ependymal ciliary beat pattern and beat frequency using high speed imaging: comparison with the photomultiplier and photodiode methods. Cilia. 2012;1: 8 doi: 10.1186/2046-2530-1-8 23351965 10.1186/2046-2530-1-8 PMC3555703

[22] Appelbe O, Bollman B, Attarwala A, Triebes L, Muniz-Talavera H, Curry D, Schmidt J. Disruption of the mouse Jhy gene causes abnormal ciliary microtubule patterning and juvenile hydrocephalus. Dev Biol. 2013;382: 172–185. doi: 10.1016/j.ydbio.2013.07.003 23906841 10.1016/j.ydbio.2013.07.003 PMC3783533

[23] Batiz L, Jimenez A, Guerra M, Rodriguez-Perez L, Toledo C, Vio K, Paez P, Perez-Figares J, Rodriguez E. New ependymal cells are born postnatally in two discrete regions of the mouse brain and support ventricular enlargement in hydrocephalus. Acta Neuropathol. 2011;121: 721–735. doi: 10.1007/s00401-011-0799-x 21311902 10.1007/s00401-011-0799-x

[24] Rubenstein JL, Rakic P. Comprehensive Developmental Neuroscience: Patterning and cell type specification in the developing CNS and PNS, 2013 ed, p. 974.

[25] Taulman P, Haycraft C, Balkovetz D, Yoder B. Polaris, a protein involved in left-right axis patterning, localizes to basal bodies and cilia. Mol Biol Cell. 2001;12: 589–599. 11251073 10.1091/mbc.12.3.589 PMC30966

[26] Lattke M, Magnutzki A, Walther P, Wirth T, Baumann B. Nuclear factor kappaB activation impairs ependymal ciliogenesis and links neuroinflammation to hydrocephalus formation. J Neurosci. 2012;32: 11511–11523. doi: 10.1523/JNEUROSCI.0182-12.2012 22915098 10.1523/JNEUROSCI.0182-12.2012 PMC6703776

[27] Castleman V, Romio L, Chodhari R, Hirst R, de Castro S, Parker K, Ybot-Gonzalez P, Emes R, Wilson S, Wallis C, Johnson C, Herrera R, Rutman A, Dixon M, Shoemark A, Bush A, Hogg C, Gardiner R, Reish O, Greene N, O'Callaghan C, Purton S, Chung E, Mitchison H. Mutations in radial spoke head protein genes RSPH9 and RSPH4A cause primary ciliary dyskinesia with central-microtubular-pair abnormalities. Am J Hum Genet. 2009;84: 197–209. doi: 10.1016/j.ajhg.2009.01.011 19200523 10.1016/j.ajhg.2009.01.011 PMC2668031

[28] Ibanez-Tallon I, Gorokhova S, Heintz N. Loss of function of axonemal dynein Mdnah5 causes primary ciliary dyskinesia and hydrocephalus. Hum Mol Genet. 2002;11: 715–721. 11912187 10.1093/hmg/11.6.715

[29] Clare D, Magescas J, Piolot T, Dumoux M, Vesque C, Pichard E, Dang T, Duvauchelle B, Poirier F, Delacour D. Basal foot MTOC organizes pillar MTs required for coordination of beating cilia. Nat Comm. 2014;5: 4888. 10.1038/ncomms5888 PMC4993237 25215410

[30] Ibanez-Tallon I, Pagenstecher A, Fliegauf M, Olbrich H, Kispert A, Ketelsen U, North A, Heintz N, Omran H. Dysfunction of axonemal dynein heavy chain Mdnah5 inhibits ependymal flow and reveals a novel mechanism for hydrocephalus formation. Hum Mol Genet. 2004;13: 2133–2141. doi: 10.1093/hmg/ddh219 15269178 10.1093/hmg/ddh219

[31] Zhou J, Yang F, Leu NA, Wang PJ. MNS1 is essential for spermiogenesis and motile ciliary functions in mice. PLoS Genet. 2012;8: e1002516 doi: 10.1371/journal.pgen.1002516 22396656 10.1371/journal.pgen.1002516 PMC3291534

[32] Mirzadeh Z, Han Y, Soriano-Navarro M, Garcia-Verdugo J, Alvarez-Buylla A. Cilia organize ependymal planar polarity. J Neurosci. 2010;30: 2600–2610. doi: 10.1523/JNEUROSCI.3744-09.2010 20164345 10.1523/JNEUROSCI.3744-09.2010 PMC2873868

[33] Ohata S, Nakatani J, Herranz-Perez V, Cheng J, Belinson H, Inubushi T, Snider WD, Garcia-Verdugo JM, Wynshaw-Boris A, Alvarez-Buylla A. Loss of Dishevelleds disrupts planar polarity in ependymal motile cilia and results in hydrocephalus. Neuron. 2014;83: 558–571. doi: 10.1016/j.neuron.2014.06.022 25043421 10.1016/j.neuron.2014.06.022 PMC4126882

[34] Ying G, Avasthi P, Irwin M, Gerstner C, Frederick J, Lucero M, Baehr W. Centrin 2 is required for mouse olfactory ciliary trafficking and development of ependymal cilia planar polarity. J Neurosci, 2014;34: 6377–6388. doi: 10.1523/JNEUROSCI.0067-14.2014 24790208 10.1523/JNEUROSCI.0067-14.2014 PMC4004820

[35] Abouhamed M, Grobe K, San I, Thelen S, Honnert U, Balda M, Matter K, Bahler M. Myosin IXa regulates epithelial differentiation and its deficiency results in hydrocephalus. Mol Biol Cell. 2009;20: 5074–5085. doi: 10.1091/mbc.E09-04-0291 19828736 10.1091/mbc.E09-04-0291 PMC2793285

[36] Baas D, Meiniel A, Benadiba C, Bonnafe E, Meiniel O, Reith W, Durand, B. A deficiency in RFX3 causes hydrocephalus associated with abnormal differentiation of ependymal cells. Eur J Neurosci. 2006;24: 1020–1030. doi: 10.1111/j.1460-9568.2006.05002.x 16930429 10.1111/j.1460-9568.2006.05002.x

[37] Didier M, Harandi M, Aguera M, Bancel B, Tardy M, Fages C, Calas A, Stagaard M. Mollgard K, Belin MF. Differential immunocytochemical staining for glial fibrillary acidic (GFA) protein, S-100 protein and glutamine synthetase in the rat subcommissural organ, nonspecialized ventricular ependyma and adjacent neuropil. Cell Tissue Res. 1986;245: 343–351. 2874885 10.1007/BF00213941

[38] Shibata T, Yamada K, Watanabe M, Ikenaka K, Wada K, Tanaka K, Inoue Y. Glutamate transporter GLAST is expressed in the radial glia-astrocyte lineage of developing mouse spinal cord. J Neurosci. 1997;17: 9212–9219. 9364068 10.1523/JNEUROSCI.17-23-09212.1997 PMC6573593

[39] Yu X, Ng C, Habacher H, Roy S. Foxj1 transcription factors are master regulators of the motile ciliogenic program. Nat Genet. 2008;40: 1445–1453. doi: 10.1038/ng.263 19011630 10.1038/ng.263

[40] Gumbiner B. Regulation of cadherin-mediated adhesion in morphogenesis. Nat Rev Mol Cell Biol. 2005;6: 622–634. doi: 10.1038/nrm1699 16025097 10.1038/nrm1699

[41] Harris T, Tepass U. Adherens junctions: from molecules to morphogenesis. Nat Rev Mol Cell Biol. 2010;11: 502–514. doi: 10.1038/nrm2927 20571587 10.1038/nrm2927

[42] Shindo M, Wada H, Kaido M, Tateno M, Aigaki T, Tsuda L, Hayashi S. Dual function of Src in the maintenance of adherens junctions during tracheal epithelial morphogenesis. Development. 2008;135: 1355–1364. doi: 10.1242/dev.015982 18305002 10.1242/dev.015982

[43] Bertocchi C, Vaman Rao M, Zaidel-Bar R. Regulation of adherens junction dynamics by phosphorylation switches. J Signal Transduct. 2012;2012: 125295 doi: 10.1155/2012/125295 22848810 10.1155/2012/125295 PMC3403498

[44] McEwen A, Escobar D, Gottardi C. Signaling from the adherens junction. Subcell Biochem. 2012:60: 171–196. doi: 10.1007/978-94-007-4186-7_8 22674072 10.1007/978-94-007-4186-7_8 PMC4031758

[45] Meng W, Takeichi M. Adherens junction: molecular architecture and regulation. Cold Spring Harb Perspect Biol. 2009;1: a002899 doi: 10.1101/cshperspect.a002899 20457565 10.1101/cshperspect.a002899 PMC2882120

[46] Halbleib J, Nelson W. Cadherins in development: cell adhesion, sorting, and tissue morphogenesis. Genes Dev. 2006;20: 3199–3214. doi: 10.1101/gad.1486806 17158740 10.1101/gad.1486806

[47] Kowalczyk A, Nanes B. Adherens junction turnover: regulating adhesion through cadherin endocytosis, degradation, and recycling. Subcell Biochem. 2012;60: 197–222. doi: 10.1007/978-94-007-4186-7_9 22674073 10.1007/978-94-007-4186-7_9 PMC4074012

[48] Heuberger J, Birchmeier W. Interplay of cadherin-mediated cell adhesion and canonical Wnt signaling. Cold Spring Harb Perspect Biol. 2010;2: a002915 doi: 10.1101/cshperspect.a002915 20182623 10.1101/cshperspect.a002915 PMC2828280

[49] Zhang J, Shemezis J, McQuinn E, Wang J, Sverdlov M, Chenn A. AKT activation by N-cadherin regulates beta-catenin signaling and neuronal differentiation during cortical development. Neural Dev. 2013;8: 7 doi: 10.1186/1749-8104-8-7 23618343 10.1186/1749-8104-8-7 PMC3658902

[50] Chilov D, Sinjushina N, Rita H, Taketo M, Makela T, Partanen J. Phosphorylated beta-catenin localizes to centrosomes of neuronal progenitors and is required for cell polarity and neurogenesis in developing midbrain. Dev Biol. 2011;357: 259–268. doi: 10.1016/j.ydbio.2011.06.029 21736876 10.1016/j.ydbio.2011.06.029

[51] Clevers H, Nusse R. Wnt/beta-catenin signaling and disease. Cell. 2012;149: 1192–1205. doi: 10.1016/j.cell.2012.05.012 22682243 10.1016/j.cell.2012.05.012

[52] Wallingford J. Planar cell polarity signaling, cilia and polarized ciliary beating. Curr Opin Cell Biol. 2010;22: 597–604. doi: 10.1016/j.ceb.2010.07.011 20817501 10.1016/j.ceb.2010.07.011 PMC2974441

[53] Kishimoto N, Sawamoto K. Planar polarity of ependymal cilia. Differentiation. 2012; 83: S86–S90. doi: 10.1016/j.diff.2011.10.007 22101065 10.1016/j.diff.2011.10.007

[54] Boutin C, Labedan P, Dimidschstein J, Richard F, Cremer H, Andre P, Yang Y, Montcouquiol M, Goffinet AM, Tissir F. A dual role for planar cell polarity genes in ciliated cells. Proc Natl Acad Sci USA. 2014;111: E3129–3138. doi: 10.1073/pnas.1404988111 25024228 10.1073/pnas.1404988111 PMC4121795

[55] Hirota Y, Meunier A, Huang S, Shimozawa T, Yamada O, Kida Y, Inoue M, Ito T, Kato H, Sakaguchi M, Sunabori T, Nakaya M, Nonaka S, Ogura T, Higuchi H, Okano H, Spassky N, Sawamoto K. Planar polarity of multiciliated ependymal cells involves the anterior migration of basal bodies regulated by non-muscle myosin II. Development. 2010;137: 3037–3046. doi: 10.1242/dev.050120 20685736 10.1242/dev.050120

[56] Ohata S, Herranz-Perez V, Nakatani J, Boletta A, Garcia-Verdugo J, Alvarez-Buylla A. Mechanosensory genes Pkd1 and Pkd2 contribute to the planar polarization of brain ventricular epithelium. J Neurosci. 2015;35: 11153–11168. doi: 10.1523/JNEUROSCI.0686-15.2015 26245976 10.1523/JNEUROSCI.0686-15.2015 PMC4524982

[57] Harding M, McGraw H, Nechiporuk A. The roles and regulation of multicellular rosette structures during morphogenesis. Development. 2014;141: 2549–2558. doi: 10.1242/dev.101444 24961796 10.1242/dev.101444 PMC4067956

[58] Ivliev A, 't Hoen P, van Roon-Mom W, Peters D, Sergeeva M. Exploring the transcriptome of ciliated cells using in silico dissection of human tissues. PLoS One. 2012;7: e35618. 10.1371/journal.pone.0035618 PMC3338421 22558177

[59] Choksi SP, Babu D, Lau D, Yu X, Roy S. Systematic discovery of novel ciliary genes through functional genomics in the zebrafish. Development. 2014;141: 3410–3419. doi: 10.1242/dev.108209 25139857 10.1242/dev.108209 PMC4199137

[60] Guerra M, Henzi R, Ortloff A, Lichtin N, Vio K, Jimenez A, Dominguez-Pinos M, Gonzalez C, Jara M, Hinostroza F, Rodriguez S, Jara M, Ortega E, Guerra F, Sival D, den Dunnen W, Perez-Figares J, McAllister J, Johanson C, Rodriguez E. Cell junction pathology of neural stem cells is associated with ventricular zone disruption, hydrocephalus, and abnormal neurogenesis. J Neuropathol Exp Neurol. 2015;74: 653–671. doi: 10.1097/NEN.0000000000000203 26079447 10.1097/NEN.0000000000000203

[61] Oliver C, Gonzalez C, Alvial G, Flores C, Rodriguez E, Batiz L. Disruption of CDH2/N-cadherin-based adherens junctions leads to apoptosis of ependymal cells and denudation of brain ventricular walls. J Neuropathol Exp Neurol. 2013;72: 846–860. doi: 10.1097/NEN.0b013e3182a2d5fe 23965744 10.1097/NEN.0b013e3182a2d5fe

[62] Yamamoto H, Maruo T, Majima T, Ishizaki H, Tanaka-Okamoto M, Miyoshi J, Mandai K Takai Y. Genetic deletion of afadin causes hydrocephalus by destruction of adherens junctions in radial glial and ependymal cells in the midbrain. PLoS One. 2013;8: e80356 doi: 10.1371/journal.pone.0080356 24236178 10.1371/journal.pone.0080356 PMC3827428

[63] Derycke L, Bracke M. N-cadherin in the spotlight of cell-cell adhesion, differentiation, embryogenesis, invasion and signalling. Int J Dev Biol. 2004;48: 463–476. doi: 10.1387/ijdb.041793ld 15349821 10.1387/ijdb.041793ld

[64] Teng J, Rai T, Tanaka Y, Takei Y, Nakata T, Hirasawa M, Kulkarni A, Hirokawa N. The KIF3 motor transports N-cadherin and organizes the developing neuroepithelium. Nat Cell Biol. 2005;7: 474–482. doi: 10.1038/ncb1249 15834408 10.1038/ncb1249

[65] Kam Y, Quaranta V. Cadherin-bound beta-catenin feeds into the Wnt pathway upon adherens junctions dissociation: evidence for an intersection between beta-catenin pools. PLoS One. 2009;4: e4580 doi: 10.1371/journal.pone.0004580 19238201 10.1371/journal.pone.0004580 PMC2640460

[66] Nakamura T, Hayashi T, Nasu-Nishimura Y, Sakaue F, Morishita Y, Okabe T, Ohwada S, Matsuura K, Akiyama T. PX-RICS mediates ER-to-Golgi transport of the N-cadherin/beta-catenin complex. Genes Dev. 2008;22: 1244–1256. doi: 10.1101/gad.1632308 18451111 10.1101/gad.1632308 PMC2335319

[67] Guirao B, Meunier A, Mortaud S, Aguilar A, Corsi J, Strehl L, Hirota Y, Desoeuvre A, Boutin C, Han Y, Mirzadeh Z, Cremer H, Montcouquiol M, Sawamoto K, Spassky N. Coupling between hydrodynamic forces and planar cell polarity orients mammalian motile cilia. Nat Cell Biol. 2014;12: 341–350. 10.1038/ncb2040 20305650

[68] Ladoux B, Nelson W, Yan J, Mege R. The mechanotransduction machinery at work at adherens junctions. Integr Biol. 2015;7: 1109–1119. 10.1039/c5ib00070j PMC4593723 25968913

[69] Etienne-Manneville S. Control of polarized cell morphology and motility by adherens junctions. Semin Cell Dev Biol. 2011;22: 850–857. doi: 10.1016/j.semcdb.2011.07.023 21839846 10.1016/j.semcdb.2011.07.023

[70] Stocker AM, Chenn A. The role of adherens junctions in the developing neocortex. Cell Adh Migr. 2015;9: 167–174. doi: 10.1080/19336918.2015.1027478 25914082 10.1080/19336918.2015.1027478 PMC4594481

[71] Hoschuetzky H, Aberle H, Kemler R. Beta-catenin mediates the interaction of the cadherin-catenin complex with epidermal growth factor receptor. J Cell Biol. 1994;127: 1375–1380. 7962096 10.1083/jcb.127.5.1375 PMC2120252

[72] Rappl A, Piontek G, Schlegel J. EGFR-dependent migration of glial cells is mediated by reorganisation of N-cadherin. J Cell Sci. 2008;121: 4089–4097. doi: 10.1242/jcs.027995 19033391 10.1242/jcs.027995

[73] Orsulic S, Huber O, Aberle H, Arnold S, Kemler R. E-cadherin binding prevents beta-catenin nuclear localization and beta-catenin/LEF-1-mediated transactivation. J Cell Sci. 1999;112: 1237–1245. 10085258 10.1242/jcs.112.8.1237

[74] Sadot E, Simcha I, Shtutman M, Ben-Ze'ev A, Geiger B. Inhibition of beta-catenin-mediated transactivation by cadherin derivatives. Proc Natl Acad Sci USA. 1998;95: 15339–15344. 9860970 10.1073/pnas.95.26.15339 PMC28044

[75] Nagaoka T, Inutsuka A, Begum K, bin Hafiz K, Kishi M. Vangl2 regulates E-cadherin in epithelial cells. Sci Rep. 2014;4: 6940 doi: 10.1038/srep06940 25373475 10.1038/srep06940 PMC4221783

[76] Lindqvist M, Horn Z, Bryja V, Schulte G, Papachristou P, Ajima R, Dyberg C, Arenas E, Yamaguchi T, Lagercrantz H, Ringstedt T. Vang-like protein 2 and Rac1 interact to regulate adherens junctions. J Cell Sci. 2010;123: 472–483. doi: 10.1242/jcs.048074 20067994 10.1242/jcs.048074 PMC2816190

