# Supplementary figures and images for: The mouse Jhy gene regulates ependymal cell differentiation and ciliogenesis
Source: PLoS One. 2017 Dec 6;12(12):e0184957. doi: 10.1371/journal.pone.0184957 (PMC5718522; doi:10.1371/journal.pone.0184957)

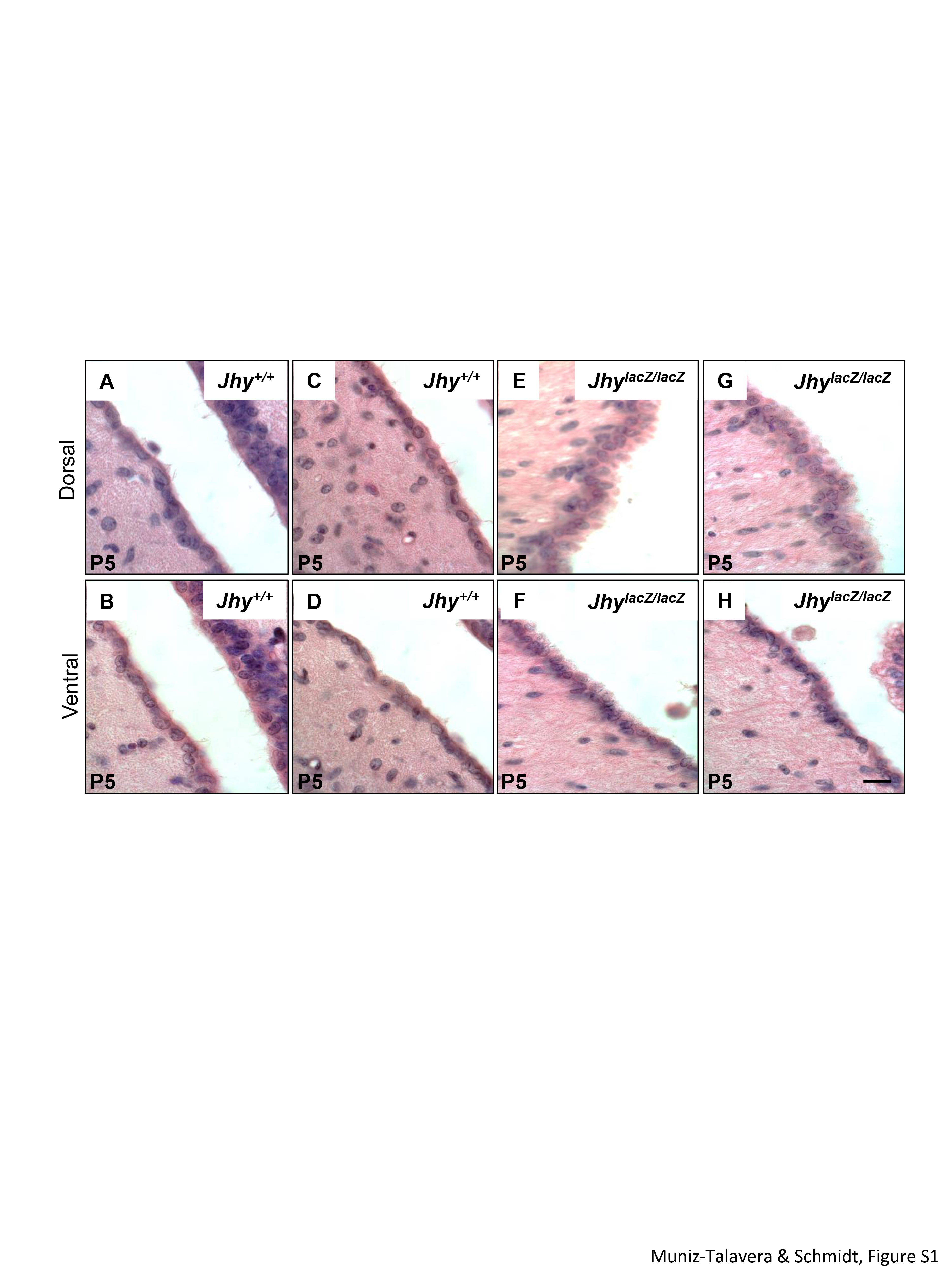

Supplement: S1 Fig — H&E staining of P5 medial wall ependyma in Jhy+/+ (A-D) and JhylacZ/lacZ (E-H). Dorsomedial (A, C) and ventromedial (B, D) ependyma in Jhy+/+ display a flattened differentiated appearance. Similar morphological characteristics are observed in ventral ependymal cells in JhylacZ/lacZ (F, H), yet undifferentiated ependymal cells are still observed in dorsally located regions (E, G). Scale bars: 20μm (A-H). (TIF) [file pone.0184957.s001.tif]

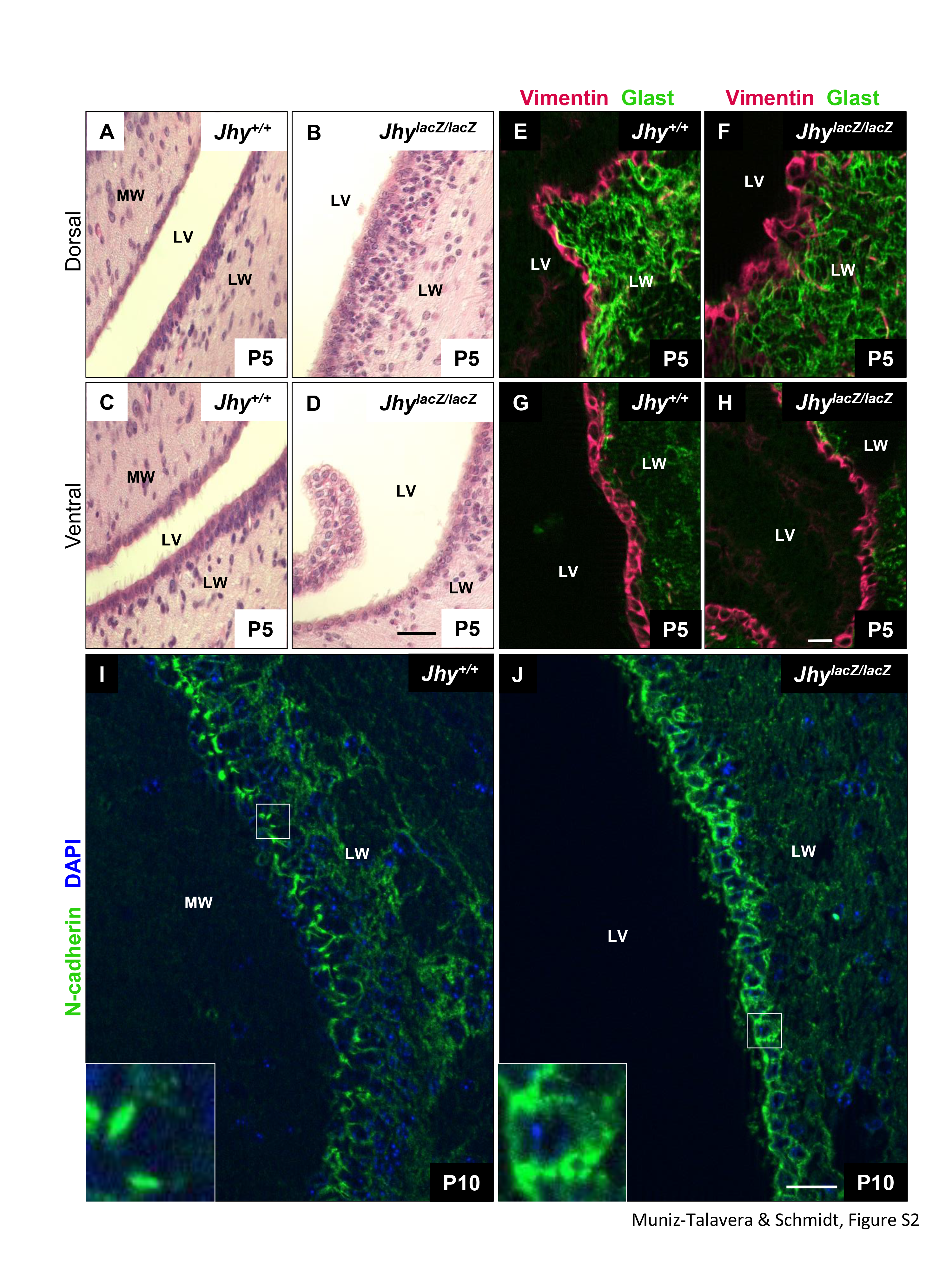

Supplement: S2 Fig — H&E staining of P5 lateral walls in both Jhy+/+ (A, C) and JhylacZ/lacZ (B, D) animals show undifferentiated cuboidal ependyma in both dorsal (A, B) and ventral (C, D) regions of the lateral wall. Lateral wall sections were used for IF for Vimentin (pink) and Glast (green) from Jhy+/+ (E, G) and JhylacZ/lacZ (F, H) brains. In both Jhy+/+ (E, G) and JhylacZ/lacZ (F, H), both dorsal (E, F) and ventral (G, H) cells were Glast(-)Vimentin(+). N-cadherin IF (green) in P10 brain shows normal apicolateral localization in Jhy+/+ (I, inset), while JhylacZ/lacZ lateral wall ependyma display abnormal basolateral N-cadherin localization (J, inset). CP, choroid plexus; MW, medial wall; LW, lateral wall; LV, lateral ventricle. Scale bars: 50μm (A-D); 20μm (E-H); 20μm (I-J). (TIF) [file pone.0184957.s002.tif]

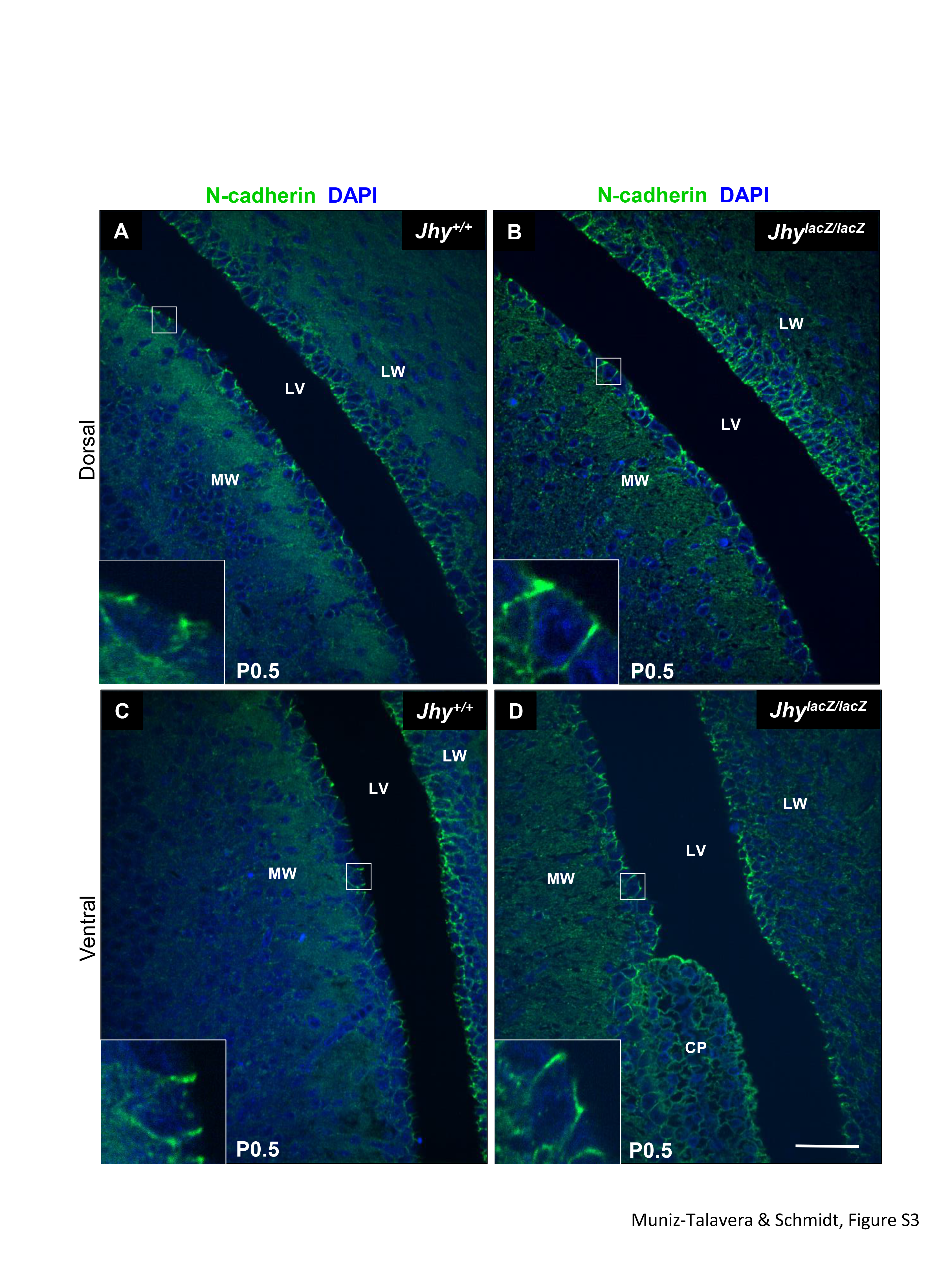

Supplement: S3 Fig — N-cadherin (green) IF in P0.5 medial wall of Jhy+/+ (A, C) and JhylacZ/lacZ (B, D). Jhy+/+ dorsal (A) and ventral (C) ependyma display normal apicolateral N-cadherin localization. JhylacZ/lacZ dorsal (B) and ventral (D) ependyma also show N-cadherin localized to the expected apicolateral position. CP, choroid plexus; MW, medial wall; LW, lateral wall; LV, lateral ventricle. Scale bars: 50μm (A-D). (TIF) [file pone.0184957.s003.tif]
